# PEGylated Heterofunctional Dendrimers Enable Multivalent Diclofenac Delivery for ROS-Driven Anticancer Activity

**DOI:** 10.1021/acsami.6c00115

**Published:** 2026-02-10

**Authors:** Arunika Singh, Natalia Sanz del Olmo, Michael Malkoch

**Affiliations:** † Department of Fibre and Polymer Technology, 7655 KTH Royal Institute of Technology, Stockholm 100 44, Sweden; ‡ Department of Organic and Inorganic Chemistry, Faculty of Sciences, Research Institute in Chemistry “Andrés M. Del Río” (IQAR), University of Alcala, Madrid 28805, Spain; § Institute “Ramón y Cajal” for Health Research (IRYCIS), Madrid 28034, Spain

**Keywords:** heterofunctional polyester dendrimers, diclofenac repurposing, PEGylation, covalent conjugation, anticancer therapeutics, cytotoxicity, ROS activity

## Abstract

Cancer drug development faces escalating costs and limited success, driving interest toward drug repurposing strategies. Diclofenac, a widely used nonsteroidal anti-inflammatory drug (NSAID) with emerging anticancer potential, exhibits poor aqueous solubility and rapid systemic clearance, limiting its chemotherapeutic suitability. Here, we engineered PEGylated heterofunctional polyester dendrimers (HFDs) as modular nanocarriers that enable controlled multivalent presentation of diclofenac through orthogonal chemistry. Diclofenac was conjugated within the dendritic interior using copper­(I)-catalyzed azide–alkyne cycloaddition (CuAAC) while peripheral PEGylation was introduced through anhydride esterification. First- and second-generation constructs, G1-(Dicl)_3_-(mPEG)_6_ and G2-(Dicl)_9_-(mPEG)_12_, assembled into amphiphilic core–shell nanostructures with hydrodynamic diameters of 170–330 nm and well-defined drug loading. G1-(Dicl)_3_-(mPEG)_6_ demonstrated the strongest therapeutic performance, reducing viability by 50–70% in MCF-7, U-87 MG, and PANC-1 cancer cells at 1–10 μM while maintaining >95% viability in noncancerous fibroblasts. This represents a >20-fold improvement in therapeutic index compared to free diclofenac. G2-(Dicl)_9_-(mPEG)_12_ displayed potent but cell-line-dependent activity, with highest efficacy in MCF-7 cells. Both dendrimers required 10–100× lower concentrations than free diclofenac to induce comparable reactive oxygen species (ROS) levels, with G1 producing 3–4-fold ROS elevation at 10 μM and G2 achieving similar induction at 0.1 μM. Mechanistic analysis confirmed ROS-mediated cytotoxicity as a key contributing pathway and correlated directly with cytotoxicity across various cancer models. These findings establish HFDs as an adaptable nanomedicine platform for repurposing clinically approved drugs, with G1 dendrimer providing the optimal balance of efficacy, selectivity, and translational potential.

## Graphical abstract

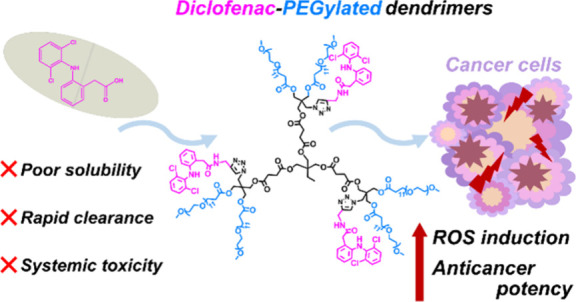

## Introduction

Cancer remains one of the most pressing global health challenges, accounting for nearly 10 million deaths in 2020.^1^ Despite major advances in treatment modalities, the development of safe and effective anticancer therapeutics remains arduous and costly, representing nearly 40% of pharmaceutical research and development expenditures.^2^ This reality has accelerated interest in drug repurposing, which redeploys commercially available drugs with established safety profiles for new therapeutic indications, offering a rapid and cost-efficient pathway to expand the oncological arsenal.^3,4^

Among repurposing candidates, NSAIDs have attracted substantial attention. Diclofenac, a widely prescribed NSAID, exerts anticancer effects through both cyclooxygenase (COX)-dependent suppression of prostaglandin-mediated tumor growth and COX-independent mechanisms, including reactive oxygen species (ROS) induction, metabolic modulation, angiogenesis inhibition, and disruption of oncogenic signaling.^5–9^ However, diclofenac suffers from major physicochemical and pharmacokinetic limitations: the neutral form exhibits extremely poor aqueous solubility (<5 mg/L), while the clinically used sodium salt undergoes rapid clearance with a plasma half-life of approximately 2 h.^10^ These limitations necessitate frequent high-dose administration, exacerbating gastrointestinal and cardiovascular toxicities.^11^ While nanocarrier strategies including poly­(lactic-*co*-glycolic acid)^12^ (PLGA) nanoparticles, micelles,^13^ and liposomes^14^ have improved diclofenac’s solubility and circulation times, encapsulation-based systems often suffer from low drug loading, burst release, and poor reproducibility, undermining their clinical translation potential.^15^ Nevertheless, diclofenac’s multifaceted anticancer activity, extensive clinical history, and favorable cost profile continue to make it an attractive candidate for oncological repurposing, provided that its delivery challenges can be effectively addressed.

Dendrimers offer a promising alternative due to their monodisperse architecture, precise molecular weight control, and tunable surface functionality.^16–19^ Among dendrimer families, poly­(amidoamine) (PAMAM)^20,21^ and aliphatic polyester dendrimers derived from 2,2-bis­(methylol)­propionic acid (bis-MPA)^16,18,22^ are most widely studied. PAMAM dendrimers offer abundant surface amines but exhibit poor biodegradability and dose-dependent cytotoxicity,^23,24^ whereas bis-MPA dendrimers present favorable biodegradability and biocompatibility.^25^ PAMAM dendrimers have been employed for noncovalent diclofenac encapsulation to improve solubility and transdermal permeation, however, these systems were not designed for oncology and suffer from uncontrolled release and low loading.^26,27^ Covalent conjugation strategies have shown enhanced pharmacokinetic stability and controlled release for several anticancer agents, including paclitaxel, gallic acid, and berberine, yet covalent diclofenac-dendrimer conjugates remain unexplored for cancer therapy.^28–31^ A fundamental limitation of both PAMAM and traditional bis-MPA dendrimers is their homofunctionality: reactive groups are confined to the periphery, causing drug payloads and functional modifications to compete for the same sites, limiting both loading capacity and functional diversity.^18^

To address these challenges, our group recently reported a proof-of-concept study demonstrating the covalent conjugation of diclofenac to HFDs.^32^ However, the generation-dependent influence of these HFDs on biological performance, particularly when combined with peripheral PEGylation, remains unexplored. This represents a significant gap at the convergence of drug repurposing, macromolecular engineering, and targeted nanomedicine.

HFDs overcome these limitations by incorporating orthogonal functionalities within distinct scaffold regions, enabling independent modification of the interior and periphery.^32–34^ This architectural advance unlocks unprecedented control over payload number, location, and spatial distribution. The AB_2_C-type HFDs constructed from a BHP-diol monomer exemplify this strategy, featuring dense internal azides for cargo conjugation via CuAAC.^32,34^ Beyond its synthetic efficiency and bioorthogonality, CuAAC generates 1,2,3-triazole linkages that are increasingly recognized as structurally advantageous motifs in anticancer drug design. Recent studies have shown that triazole-containing small molecules and conjugates exhibit cytotoxic activity across diverse cancer cell lines through mechanisms including kinase inhibition and oxidative stress induction.^35–38^ In this context, the triazole linkages formed within the HFD interior serve not only as chemically robust connectors but may also provide auxiliary biological relevance, complementing the conjugated diclofenac payload. In parallel, peripheral hydroxyl groups enable esterification with entities such as methoxy poly­(ethylene glycol) propionic acid^32,39^ (mPEG_11_-PA) to form acid-labile ester linkages.

PEGylation is pivotal for optimizing dendrimer-based therapeutics, improving solubility while forming a hydrophilic corona that reduces protein adsorption and reticuloendothelial clearance, thereby prolonging blood circulation and promoting tumor accumulation via the enhanced permeability and retention (EPR) effect.^40,41^ In homofunctional scaffolds, PEGylation and drug conjugation are mutually exclusive processes, limiting overall drug loading.^39^ In contrast, HFDs enable simultaneous PEGylation and high drug loading, offering enhanced pharmacological performance.

This study reports the rational design, synthesis, and biological evaluation of first- and second-generation PEGylated HFDs, with the second-generation construct incorporating up to nine covalently attached diclofenac moieties. Using AB_2_C type BHP-diol polyester scaffolds,^32,34^ diclofenac was conjugated to the interior azide groups via CuAAC, while the periphery was decorated with PEG through anhydride-based esterification reactions. The resulting amphiphilic core–shell nanostructures provided high diclofenac payloads, enhanced aqueous solubility, and exhibited generation-dependent assembly behavior. Consequently, the role of these new dendritic systems in modulating the anticancer therapeutic performance of diclofenac was assessed through comparison with free diclofenac sodium (Dicl-Na) and a linear diclofenac-mPEG conjugate (Dicl-mPEG) across different cancer and noncancer cell lines, including mechanistic evaluation of intracellular ROS generation to elucidate the structure–activity relationships.

## Experimental Section

### Materials

All solvents and reagents were sourced from Sigma-Aldrich and used without further modification unless stated otherwise. Silica gel for column chromatography was obtained from ICN SiliTech (ICN Biomedicals GmbH, Eschwege, Germany). Dicl-Na was purchased from LGC Standards (Toronto, Canada).

Cell culture media and supplements, including Dulbecco’s Modified Eagle Medium (DMEM), fetal bovine serum (FBS), and penicillin/streptomycin antibiotic mixture, were acquired from Thermo Fisher Scientific. The following cell lines were purchased from the American Type Culture Collection (ATCC): hDF (human dermal fibroblasts), U-87 MG (human glioblastoma), PANC-1 (human pancreatic adenocarcinoma) and MCF-7 (human breast cancer). All cell lines were cultured in DMEM supplemented with 10% FBS and penicillin/streptomycin (100 units per mL^–1^ and 100 μg mL^–1^, respectively), and maintained at 37 °C with 5% CO_2_ atmosphere.

### Synthetic Protocols

All synthetic protocols are described in the Supporting Information.

### Characterization Methods

#### Nuclear Magnetic Resonance (NMR)

NMR spectroscopy was carried out on a Bruker Avance III 400 MHz spectrometer. ^1^H NMR spectra were recorded at 400 MHz with a spectral window of 20 ppm, a relaxation delay of 1 s, and 16 scans, utilizing automatic locking and shimming. ^13^C NMR spectra were acquired at 101 MHz using a spectral window of 240 ppm, a relaxation delay of 2 s, between 256 to 1024 scans. Diffusion-ordered spectroscopy (DOSY) NMR was performed at 25 °C with a spectral width of 12 ppm, transmitter frequency offset of 5 ppm, prescan delay of 6.5 μs, relaxation delay of 3 s, and 16 scans. Samples were prepared in deuterated solvents (CDCl_3_ and (CD_3_)_2_CO). ^1^H NMR spectra were referenced to residual solvent peaks of CDCl_3_ (δ 7.26 ppm) and (CD_3_)_2_CO (δ 2.05 ppm), and ^13^C NMR spectra to CDCl_3_ (δ 77.16 ppm) and (CD_3_)_2_CO (δ 29.84 ppm). Signal assignments were supported by two-dimensional NMR experiments (HSQC and HMBC). All spectra were processed and analyzed using MestReNova v14.2.0–26256 (Mestrelab Research S.L., 2020).

#### Matrix-Assisted Laser Desorption/Ionization Time-of-Flight (MALDI-TOF) Mass Spectrometry

MALDI-TOF was performed using a Bruker UltrafleXtreme MALDI-TOF mass spectrometer (Bruker Daltonics, Bremen, Germany) equipped with a SmartbeamII laser (355 nm, UV), operated in positive ion mode. Calibration was achieved using SpheriCal calibrants (Polymer Factory, Sweden). The spectra were acquired in reflector mode with an acceleration voltage of 25 kV and a reflector voltage of 26.3 kV, with laser intensity adjusted between 50 and 100% for high-resolution spectra. Data acquisition and analysis were conducted using FlexControl and FlexAnalysis Version 3.4 (Bruker Daltonics). Matrices, including 2,5-dihydroxybenzoic acid (DHB) and trans-2-[3-(4-*tert*-butylphenyl)-2-methyl-2-propenylidene]­malononitrile (DTCB), were prepared in tetrahydrofuran (THF) at 20 mg/mL. The samples were spotted onto MPT 384 ground steel TF target plate (Bruker Daltonics) by sequential deposition of 1 μL analyte followed by 2 μL matrix.

#### Size Exclusion Chromatography (SEC)

SEC was performed on a TOSOH EcoSEC HLC-8320GPC system equipped with an EcoSEC RI detector and three PSS PFG 5 μm columns (Microguard, 100 Å, and 300 Å; PSS GmbH) with a molecular weight resolving range of 300–100000 Da. Dimethylformamide (DMF) containing 0.01 M lithium bromide (LiBr) was used as the mobile phase at a flow rate of 0.2 mL min^–1^ and maintained at 35 °C. SEC samples (2–4 mg mL^–1^) were prepared from the Dicl-mPEG conjugate, diclofenac functional dendritic precursors and diclofenac-PEGylated dendrimers. The calibration was performed using narrow linear poly­(methyl methacrylate) (PMMA) standards purchased from PSS GmbH. Toluene was used as an internal standard to correct for flow rate fluctuations. The data were processed using WinGPC Unity software (v7.2) and the graphs were plotted in Origin (version 9.1.0 Sr1).

#### Fourier Transform Infrared Spectroscopy (FTIR)

FTIR spectroscopy was carried out on a PerkinElmer instrument equipped with an ATR module. All samples were dried under vacuum prior to analysis and measured over the range of 600–4000 cm^–1^ at a resolution of 4 cm^–1^, averaging 16 scans. The baseline correction and normalization were performed using Spectrum IR software (v10.5.1, PerkinElmer).

#### Dynamic Light Scattering (DLS)

DLS measurements were performed on a Malvern Zetasizer Nano ZS at 37 °C in phosphate-buffered saline (PBS) with dendritic concentrations ranging from 40 to 500 μM. Samples were equilibrated for 2 min at the measurement temperature prior to analysis. Each data set represents the average of at least three independent samples with each sample measurement consisting of five replicates (10 runs per replicate). The data were processed using ZS Xplorer software (v3.31, Malvern Panalytical).

#### Cytotoxicity Assessment

The cytotoxicity assessment of the diclofenac drug derivatives and diclofenac-PEGylated dendrimers was conducted using the Alamar Blue assay. The various cell lines including hDF, MCF-7, U-87 MG, and PANC-1 were cultured in DMEM supplemented with 10% FBS and 100 units mL^–1^ of penicillin and streptomycin at 37 °C in an incubator containing 5% CO_2_. The cells were detached with trypsin, seeded in 96-well plates at a density of 5 × 10^4^ cells per 100 μL medium, and allowed to adhere overnight. The following day, the culture medium was replaced with fresh DMEM containing test compounds at concentrations ranging from 0.1 to 320 μM. The cells were incubated for 24 and 72 h treatment periods respectively, with each concentration tested in triplicate. After the treatment period, the medium was replaced with 100 μL of a 90:10 mixture of DMEM and Alamar Blue reagent, followed by a 4 h of incubation. The fluorescence intensity was recorded using a Tecan Infinite M200 Pro plate reader at excitation/emission wavelengths of 560/590 nm. All experiments were repeated independently three times (n = 3) using freshly prepared stock solutions of drugs and dendrimers.

#### Intracellular ROS Assay

The amount of intracellular ROS generated upon exposure to different treatments was determined using the Fluorometric Intracellular ROS Kit (MAK145). The experimental setup, including cell culture conditions and seeding density (cell seeding in 96 well black-walled, clear bottom plates), was identical to that described for the cytotoxicity assay. The different cell lines: hDF, U-87 MG, PANC-1, and MCF-7, were similarly treated with diclofenac drug derivatives and diclofenac-PEGylated dendrimers for 24 and 72 h incubation periods, at relevant concentrations selected based on their cytotoxicity profiles. Following incubation, the culture medium was removed and replaced with 100 μL of the ROS detection master mix (ROS detection reagent + assay buffer). After a 1 h of incubation at 37 °C, the fluorescence intensity was measured using a Tecan Infinite M200 Pro plate reader at excitation/emission wavelengths of 520/605 nm. Only viable cells are capable of generating ROS; therefore, ROS levels were normalized to the percentage of viable cells obtained from the cytotoxicity assays. This ensures that the measured ROS reflects the activity per living cell rather than being influenced by cell death. All experiments were repeated independently two to three times (n = 2–3) using freshly prepared stock solutions of drugs and dendrimers. Data from both cytotoxicity and ROS assays were analyzed using Microsoft Excel and plotted using Origin (version 9.1.0 Sr1).

## Results and Discussion

### Synthesis and Characterization of Diclofenac-Functionalized PEGylated HFDs

The selection of diclofenac as the therapeutic payload is motivated by its emerging potential as an anticancer agent, as indicated in the Repurposing Drugs in Oncology (ReDO) initiative.^5^ Unlike other NSAIDs, diclofenac exerts unique anticancer mechanisms, including modulation of oncogenic pathways and tumor metabolism.^6,7^ Although repurposing diclofenac as an anticancer agent presents a compelling therapeutic opportunity beyond its conventional anti-inflammatory use, its clinical translation is constrained by inherent physicochemical limitations, including poor aqueous solubility and a short plasma half-life.^10^ These challenges highlight the need for advanced delivery systems capable of improving solubility, extending circulation time, and enhancing therapeutic safety. To harness diclofenac’s anticancer properties and circumvent solubility- and toxicity related–related limitations, we designed a sophisticated dendritic construct that covalently incorporates multiple diclofenac moieties and mPEG chains within a single monodisperse framework (Figure 1).

**Figure 1:**
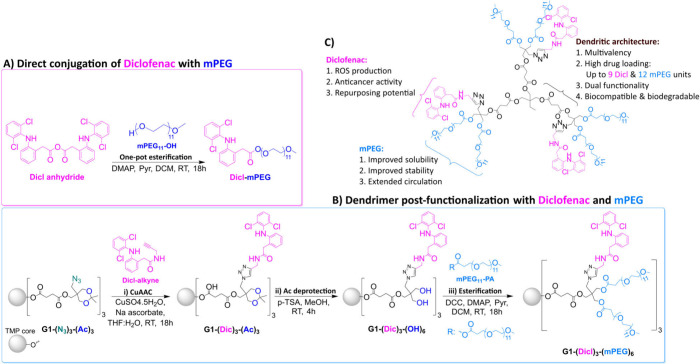
Schematic representation of the synthetic strategy toward (A) Dicl-mPEG and (B) diclofenac-PEGylated dendrimers, illustrated using the first-generation derivatives as examples. (C) Key structural and functional features of the newly synthesized diclofenac-PEGylated dendrimers.

To accomplish this, we employed a previously developed HFD platform based on an AB_2_C-type BHP-diol monomer.^32,34^ This architecture enables spatially orthogonal functionalization, with internal azide groups allocated exclusively for diclofenac conjugation^32^ and peripheral hydroxyl groups available for controlled PEGylation, thereby yielding a bifunctional therapeutic system (Figure 1C). Unlike PAMAM and bis-MPA dendrimers, which rely solely on homofunctional peripheral groups for drug attachment^18,42–44^ and are therefore limited in payload capacity, this heterofunctional design provides precise control over the number, location, and spatial arrangement of therapeutic entities within a single scaffold. The resulting diclofenac-PEGylated dendrimers directly address key barriers that have hindered diclofenac’s clinical translation as an anticancer agent. By segregating diclofenac within the dendritic interior and PEG chains on the periphery, the system achieves a balance between hydrophobic drug loading and hydrophilic stealth properties. The multivalency of the biodegradable polyester backbone enables high payload densities, while the PEG corona improves aqueous solubility, extends circulation, and facilitates passive tumor accumulation through the EPR effect.^40,41,45^ Collectively, these features overcome pharmacokinetic and solubility limitations and provide a modular, versatile platform with potential for future applications, including combination therapy, targeted delivery, and imaging.

Figure 1B outlines the synthetic pathway for obtaining diclofenac-PEGylated HFDs. To maintain consistency in the description of the synthesized dendrimers, the notation G_n_-(internal groups)_*x*_-(external groups)_*y*_ was adopted. In this format, G_n_ where n denotes the dendrimer generation, x specifies the number of internal diclofenac moieties (Dicl), and y represents the number of peripheral hydroxyl groups or mPEG chains, respectively. Since the trimethylolpropane (TMP) core remains identical across all generations, it is not explicitly included in the nomenclature. In the first step, azide-functional acetonide-protected dendrimers underwent CuAAC with alkyne-functionalized diclofenac^32^ (Dicl-alkyne) under mild conditions, employing copper sulfate pentahydrate (CuSO_4_.5H_2_O) as the catalyst and sodium ascorbate (Na ascorbate) as the reducing agent in a 1:1 THF:H_2_O mixture overnight. Subsequent purification by sequential washes with 0.5% w/w EDTA solution and passage through silica plugs efficiently removed residual CuSO_4_.5H_2_O, Na ascorbate, and unreacted Dicl-alkyne. The resulting intermediates G1-(Dicl)_3_-(Ac)_3_ ^32^ and G2-(Dicl)_9_-(Ac)_6_ were obtained as colorless oils in near-quantitative yields. This approach exemplifies the exceptional drug-loading capacity (up to nine diclofenac molecules in the G2 dendrimer), which was achieved through a single-step reaction enabled by precise azide stoichiometry and CuAAC efficiency.

Next, diclofenac-functionalized acetonide (Ac) precursors were deprotected using 12 wt % p-toluenesulfonic acid (p-TSA) in excess methanol (MeOH) at room temperature for 4 h to activate the hydroxyl groups on the dendritic periphery. The acid was then neutralized with equimolar quantities of pyridine to form pyridinium p-toluenesulfonate (PPTS), after which the crude products were dissolved in DCM and washed sequentially with NaHCO_3_ and brine to remove residual p-TSA and PPTS. The resulting diclofenac-functional hydroxyl derivatives were obtained as colorless oils, with G1-(Dicl)_3_-(OH)_6_ achieved in excellent yield (97%) and G2-(Dicl)_9_-(OH)_12_ in moderate yield (50%). The presence of peripheral hydroxyl groups yielded reactive sites for subsequent PEGylation in the final step, enabling the incorporation of a hydrophilic corona around the dendritic scaffold. This was facilitated by anhydride-based esterification of the peripheral hydroxyl groups with mPEG_11_-PA,^32,39^ activated in situ with N,N′-dicyclohexylcarbodiimide (DCC) in the presence of 4-dimethylaminopyridine (DMAP) and pyridine as bases. The reaction mixture was stirred overnight, and the crude product was purified by three successive precipitations in cold ether to remove excess mPEG_11_-PA, mPEG_11_-anhydride,^32,39^ and residual bases. The resulting PEGylated dendrimers, G1-(Dicl)_3_-(mPEG)_6_ and G2-(Dicl)_9_-(mPEG)_12_, were isolated in good yields (66–70%) as viscous, colorless oils that were readily soluble in water. By sequestering hydrophobic diclofenac moieties within the dendrimer’s interior and hydrophilic PEG on the dendritic exterior, this synthetic strategy yields a distinct core–shell structure comprised of a drug-rich core enveloped by a functional hydrophilic periphery. This design was intended to mitigate premature drug release, often observed for physically encapsulated formulations,^12–14,26,27^ resulting in enhanced payload stability and more reliable drug delivery.

To systematically evaluate the dendritic architecture contributions toward anticancer efficacy, we synthesized a control system in which diclofenac was directly conjugated to mPEG_11_–OH through a one-pot esterification protocol, analogous to the anhydride-based esterification described earlier (Figure 1A). In this modified approach, the catalytic bases and mPEG_11_–OH were added directly to the same reaction mixture containing the diclofenac anhydride (2 h activation with DCC) without the removal of dicyclohexylurea (DCU) byproduct the next day. The crude product was concentrated and purified by a short plug of silica gel, eluting from 20:80 EtOAc:heptane to 90:10 EtOAc:MeOH, to yield the pure Dicl-mPEG conjugate. This amphiphilic linear drug conjugate served as a critical control, enabling direct comparison with Dicl-Na and the diclofenac-PEGylated dendritic systems. This design facilitated the elucidation of structure–activity relationships by enabling direct comparison with diclofenac-PEGylated systems, thereby determining whether the enhanced anticancer activity arose solely from the drug-mPEG combination or whether the dendritic scaffold played a decisive role in potentiating diclofenac’s anticancer efficacy *in vitro*.

All the diclofenac-PEGylated dendrimers, along with their intermediates and precursors, as well as the Dicl-mPEG control synthesized as outlined in Figure 1, were comprehensively characterized using MALDI-TOF (Figure 2A, Figures S13–S15), SEC (Figure 2B, Figure S11, Figure S12), NMR spectroscopy (Figure 2C, Figures S1–S9), and FTIR (Figure S10). Collectively, these complementary techniques confirmed successful dual functionalization, high structural precision, purity and the monodisperse nature of the synthesized constructs.

**Figure 2:**
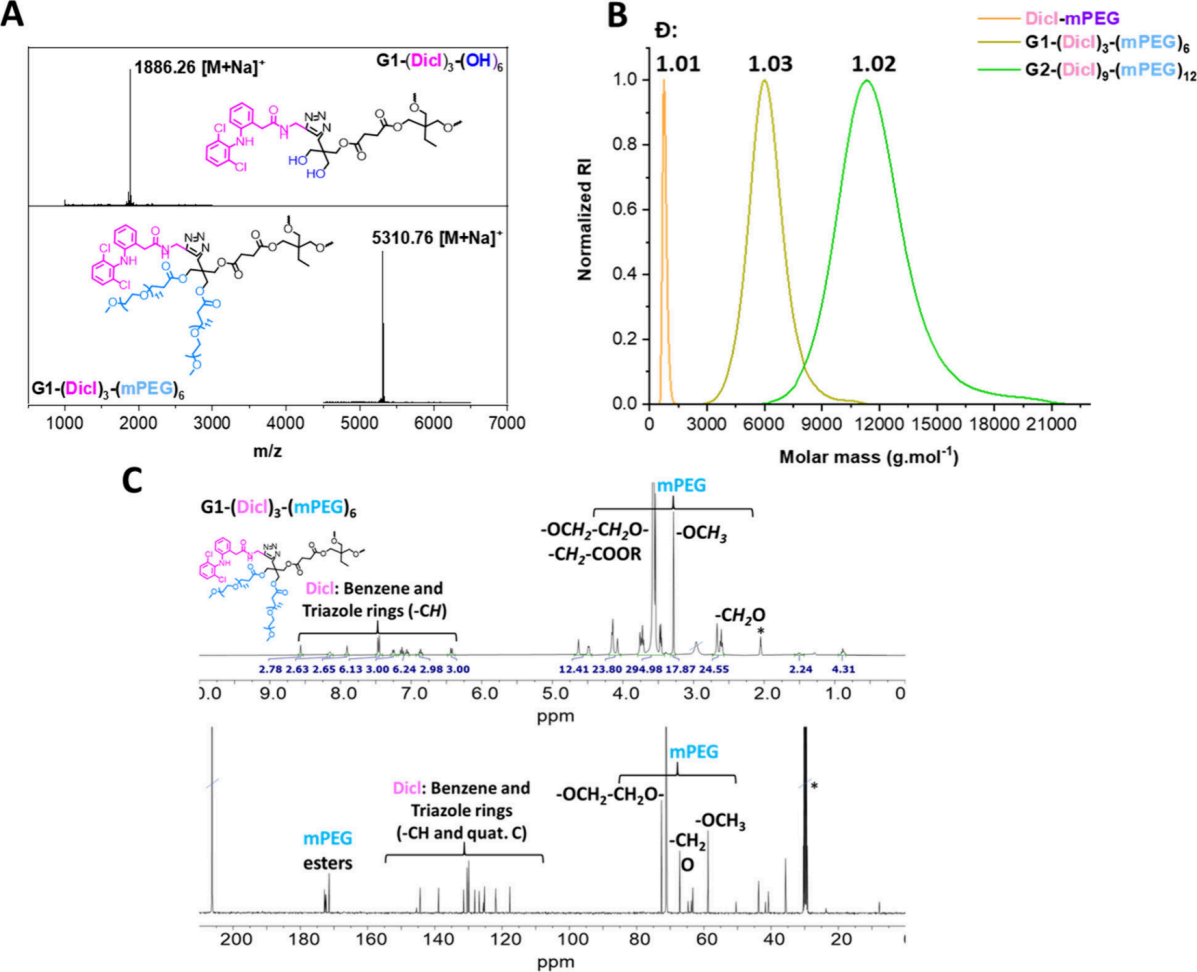
(A) Stacked MALDI-TOF spectra of G1-(Dicl)_3_-(OH)_6_ (top) and G1-(Dicl)_3_-(mPEG)_6_ (bottom) in DCTB. (B) SEC overlay of Dicl-mPEG, G1-(Dicl)_3_-(mPEG)_6_ and G2-(Dicl)_9_-(mPEG)_12_. (C) Stacked ^1^H NMR and ^13^C NMR spectra of G1-(Dicl)_3_-(mPEG)_6_ in (CD_3_)_2_CO (*).

To begin with, and consistent with previously reported studies,^32,34^ the first step was to confirm CuAAC functionalization of the azide-containing precursors with the alkyne-modified therapeutic entity, Dicl-alkyne.^32^ This was verified by FTIR analysis of the dried samples, where successful postfunctionalization was evidenced by the complete disappearance of the characteristic azide stretch at 2101 cm^–1^ (Figure S10). MALDI-TOF (Figure 2A) provided clear evidence of stepwise dendrimer functionalization, showing distinct molecular weight shifts between the precursor (G1-(Dicl)_3_-(OH)_6_) and product (G1-(Dicl)_3_-(mPEG)_6_) from 1886.26 to 5310.76 Da, indicating the addition of six m-PEG chains. This precise mass increase confirmed the homogeneity and structural integrity of the final G1 dendrimer. SEC analysis (Figure 2B) further confirmed the monodispersity of the postfunctionalized constructs by exhibiting narrow dispersity indices (Đ ∼ 1.01–1.03) for Dicl-mPEG, G1-(Dicl)_3_-(mPEG)_6_, and G2-(Dicl)_9_-(mPEG)_12_, respectively. ^1^H and ^13^C NMR spectroscopy (Figure 2C) provided complementary structural validation. The ^1^H NMR spectra of G1-(Dicl)_3_-(mPEG)_6_ showed distinct aromatic proton signals between 7.1 and 7.6 ppm attributed to diclofenac’s dichloroaniline rings, together with the diagnostic triazole proton at 7.8 ppm, confirming successful internal conjugation with Dicl-alkyne. PEGylation was verified by the appearance of the characteristic methylene envelope at 3.6 ppm (−OCH_2_–CH_2_O- repeats), the terminal methoxy signal at 3.3 ppm, a distinct methylene signal adjacent to the ester linkage at 2.6 ppm and new ester carbonyl peaks in the ^13^C NMR spectrum at 171 ppm. Additional carbon signals at ∼ 70 ppm (mPEG methylene carbons) and ∼ 59 ppm (terminal methoxy groups) corroborated the complete peripheral functionalization with mPEG. The ^1^H NMR signal integration corresponded precisely to the designated structure incorporating 3 diclofenac moieties and 6 mPEG chains, with DOSY-NMR (Figure S3) revealing single, uniform diffusion coefficients, excluding the presence of free mPEG_11_-PA or partially functionalized species.

Taken together, FTIR, MALDI-TOF, SEC, and NMR analyses established that the synthesized dendritic conjugates were structurally precise, fully functionalized, and monodisperse, with composition matching exactly the intended architectures. All the diclofenac-dendrimer intermediates (Ac and OH derivatives), diclofenac-PEGylated constructs (G2 dendrimer) and the control (Dicl-mPEG) were similarly validated to confirm their purity and structural integrity, with extended characterization provided in the Supporting Information.

DLS measurements were performed at 37 °C in filtered PBS at a concentration of 160 μM to assess the hydrodynamic properties of diclofenac-PEGylated dendrimers. The constructs displayed a systematic, generation-dependent increase in hydrodynamic diameter: Dicl-mPEG (173 ± 3.0 nm) < G1-(Dicl)_3_-(mPEG)_6_ (280 ± 6.3 nm) < G2-(Dicl)_9_-(mPEG)_12_ (327 ± 3.5 nm) (Figure 3A, B). These Z-average values are considerably larger than those typically reported for unmodified dendrimers, which generally range from 1 to 14 nm depending on generation.^46^ The observed hydrodynamic diameters (170–330 nm) substantially exceed the theoretical molecular dimensions of individual dendrimers, indicating that the functionalized constructs do not exist as isolated macromolecules under the measurement conditions. Analysis of intensity-weighted (D_i_), volume-weighted (D_v_), and number-weighted (D_n_) distributions showed variations ranging from 78.9 to 342.7 nm, reflecting the different weighting factors applied to each distribution type.

**Figure 3:**
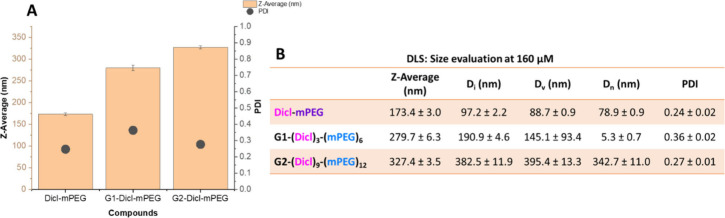
DLS analysis of Dicl-mPEG, G1-(Dicl)_3_-(mPEG)_6_ and G2-(Dicl)_9_-(mPEG)_12_ in PBS at 160 μM and 37 °C. (A) Z-average size (nm, bars, left axis) and polydispersity indices (PDI, circles, right axis) at 160 μM. (B) Summary of hydrodynamic diameters for representative constructs, including intensity- (D_i_), volume- (D_v_), and number-weighted (D_n_) diameters. Mean values accompanied by standard deviation (SD), *n* ≥ 3.

The PDI values ranged between 0.24 and 0.36, exceeding the threshold for monodisperse systems (PDI < 0.1) and the typical criterion for polymer nanoparticles (PDI < 0.2).^38^ The linear Dicl-mPEG control exhibited the lowest PDI (0.24 ± 0.02), whereas dendritic constructs showed broader size distributions (G1:0.36 ± 0.02; G2:0.27 ± 0.01), reflecting increased structural complexity and the potential for aggregation in these amphiphilic systems.

### Cytotoxicity Evaluation and ROS Generation

After establishing the structural integrity and hydrodynamic properties of the diclofenac-PEGylated dendritic constructs, their biological performance was evaluated to validate the anticancer potential of this multifunctional platform. The HFDs were designed to address diclofenac’s pharmacokinetic limitations^10,47^ while amplifying its anticancer efficacy through controlled multivalent drug presentation. The combination of precise covalent drug loading and peripheral PEGylation was expected to improve cellular uptake, reduce systemic toxicity, and enhance selectivity toward cancer cells compared to free diclofenac. To evaluate these hypotheses and establish structure–activity relationships, cytotoxicity was assessed using the Alamar Blue assay in both noncancerous (hDF) and cancerous (MCF-7, U-87 MG, and PANC-1) cell lines following 24 h (Figure S17) and 72 h (Figure 4, Figure S18) treatment periods across a concentration range of 0.1–80 μM. In parallel, intracellular ROS generation was examined to provide complementary mechanistic insight into diclofenac-induced anticancer activity.

**Figure 4:**
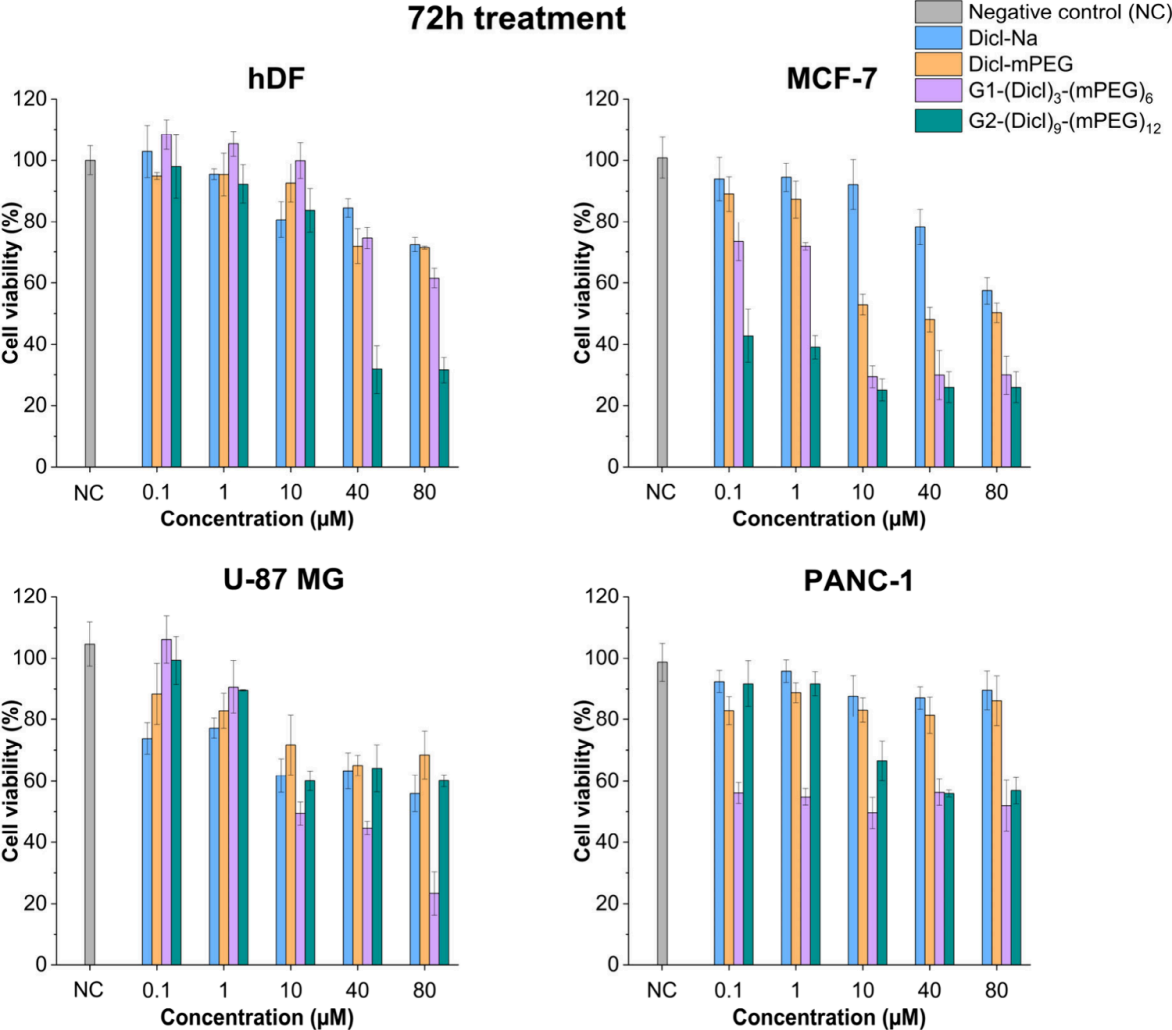
Dose–response curves showing cell viability (%) versus compound concentration (0.1–80 μM) for hDF, MCF-7, U-87 MG, and PANC-1 cells, respectively, after 72 h exposure to Dicl-Na, Dicl-mPEG, G1-(Dicl)_3_-(mPEG)_6_, and G2-(Dicl)_9_-(mPEG)_12_. Data are presented as mean values ± SD (*n* = 3).

Initial screening at 24 h revealed minimal cytotoxicity across all tested constructs (Figure S17), indicating that this time frame was insufficient for the dendritic systems to exert their full therapeutic effect. This observation reflects the complex sequence of cellular uptake, intracellular trafficking, drug release, and target engagement characteristic of controlled-release macromolecular systems.^47^ Consequently, a 72 h treatment period was assessed for the comprehensive determination of structure–activity relationships.

The 72-h cytotoxicity evaluation revealed distinct concentration-dependent anticancer effects with pronounced generation- and cell line-specific activity profiles (Figure 4). At the lowest tested concentration (0.1 μM), both dendritic constructs exhibited measurable cytotoxic effects, whereas the control compounds remained largely inactive. Specifically, G2-(Dicl)_9_-(mPEG)_12_ reduced MCF-7 cell viability to approximately 45%, while G1-(Dicl)_3_-(mPEG)_6_ showed comparable potency in PANC-1 cells (approximately 50% viability). In contrast, both Dicl-Na and Dicl-mPEG produced negligible effects across all cancer cell lines, while noncancerous hDF fibroblasts remained unaffected (>90% viability), confirming the selectivity of the dendritic conjugates at submicromolar concentrations.

At intermediate concentrations (1–10 μM), generation-dependent selectivity became increasingly pronounced. G1-(Dicl)_3_-(mPEG)_6_ demonstrated strong anticancer activity, reducing cell viability to approximately 30% in MCF-7 and 50% in both U-87 MG and PANC-1 cells, while maintaining excellent biocompatibility with hDF cells (>95% viability). G2-(Dicl)_9_-(mPEG)_12_ exhibited comparable efficacy in MCF-7 cells (∼30% viability) but markedly reduced activity in PANC-1 cells (∼70% viability), indicating cell type-specific architectural preferences.

At higher concentrations (40–80 μM), differential safety profiles emerged. The G1 dendrimer maintained potent anticancer effects while exhibiting favorable selectivity, with hDF cells retaining 75% and 60% viability at 40 μM and 80 μM, respectively. Conversely, the G2 dendrimer induced substantial cytotoxicity beyond 40 μM, indicating a significantly narrower therapeutic window. The drug controls remained markedly less active across the entire concentration range, confirming the synergistic enhancement achieved through multivalent presentation combined with PEGylation.

MCF-7 cells exhibited unique sensitivity to PEGylation, as the linear Dicl-mPEG conjugate achieved substantial cytotoxicity (50% viability at 10 μM) compared to Dicl-Na (93% viability), representing the only cell line where PEGylation alone significantly enhanced free drug’s activity. Remarkably, both dendrimers converged to nearly identical dose–response profiles in MCF-7 cells from 10 μM onward, indicating efficient cellular processing of both dendrimer generations in this breast cancer model. In contrast, U-87 MG and PANC-1 cancer cells displayed pronounced generation-dependent selectivity. The G1 dendrimer consistently outperformed G2 dendrimer across most concentrations, with the disparity most evident in PANC-1 cells, where the G1 dendrimer demonstrated potent activity from 0.1 μM onward while the G2 dendrimer remained largely ineffective until higher concentrations. This preferential selectivity suggests that pancreatic cancer cells possess specific cellular uptake mechanisms that strongly favor the first-generation dendrimer. The cell-line specific activity suggests potential for personalized treatment approaches, particularly given the pronounced efficacy of G1 dendrimer in PANC-1 cells, considering the well documented drug resistance and treatment challenges associated with pancreatic cancer.^40^

The differential performance between both dendrimer generations provides critical insights into multivalent drug delivery design. Despite the G2 dendrimer containing 3-fold more diclofenac molecules (9 vs 3 units), it does not consistently demonstrate superior anticancer activity, indicating that optimal therapeutic performance depends on the balance between drug loading, dendrimer size, and cellular interactions rather than simply maximizing drug content. The reduced potency of the G2 dendrimer may reflect altered cellular uptake kinetics or suboptimal drug release profiles associated with the larger, more densely loaded structure.

In summary, G1-(Dicl)_3_-(mPEG)_6_ emerges as the optimal construct, delivering consistent potent anticancer activity across diverse cancer cell lines while maintaining excellent safety profiles. The superior performance of the first-generation dendrimer, despite lower drug loading, validates the importance of balanced multivalent design in developing effective nanotherapeutics and establishes a strong foundation for advancing diclofenac-PEGylated dendrimers toward clinical development.

To elucidate the molecular basis of the observed cytotoxicity, intracellular ROS generation was assessed at 24 and 72 h for both dendritic constructs (Figure 5) and the control drugs Dicl-Na and Dicl-mPEG (Figure S19). The concentrations for ROS evaluation were strategically selected based on the distinct cytotoxicity profiles of each compound, focusing on therapeutically relevant ranges that corresponded to significant biological activity in the viability assays. Consequently, the concentrations displayed on the *x*-axes vary among G1-(Dicl)_3_-(mPEG)_6_, G2-(Dicl)_9_-(mPEG)_12_, and the control drug derivatives, enabling direct correlation between oxidative stress and loss of cell viability for each construct. Comprehensive ROS data across broader concentration ranges are provided in the Supporting Information (Figure S19, Figure S20). This design allowed mechanistic insight into one of the key pathways contributing to the anticancer effects of the diclofenac-PEGylated dendritic platform.

**Figure 5:**
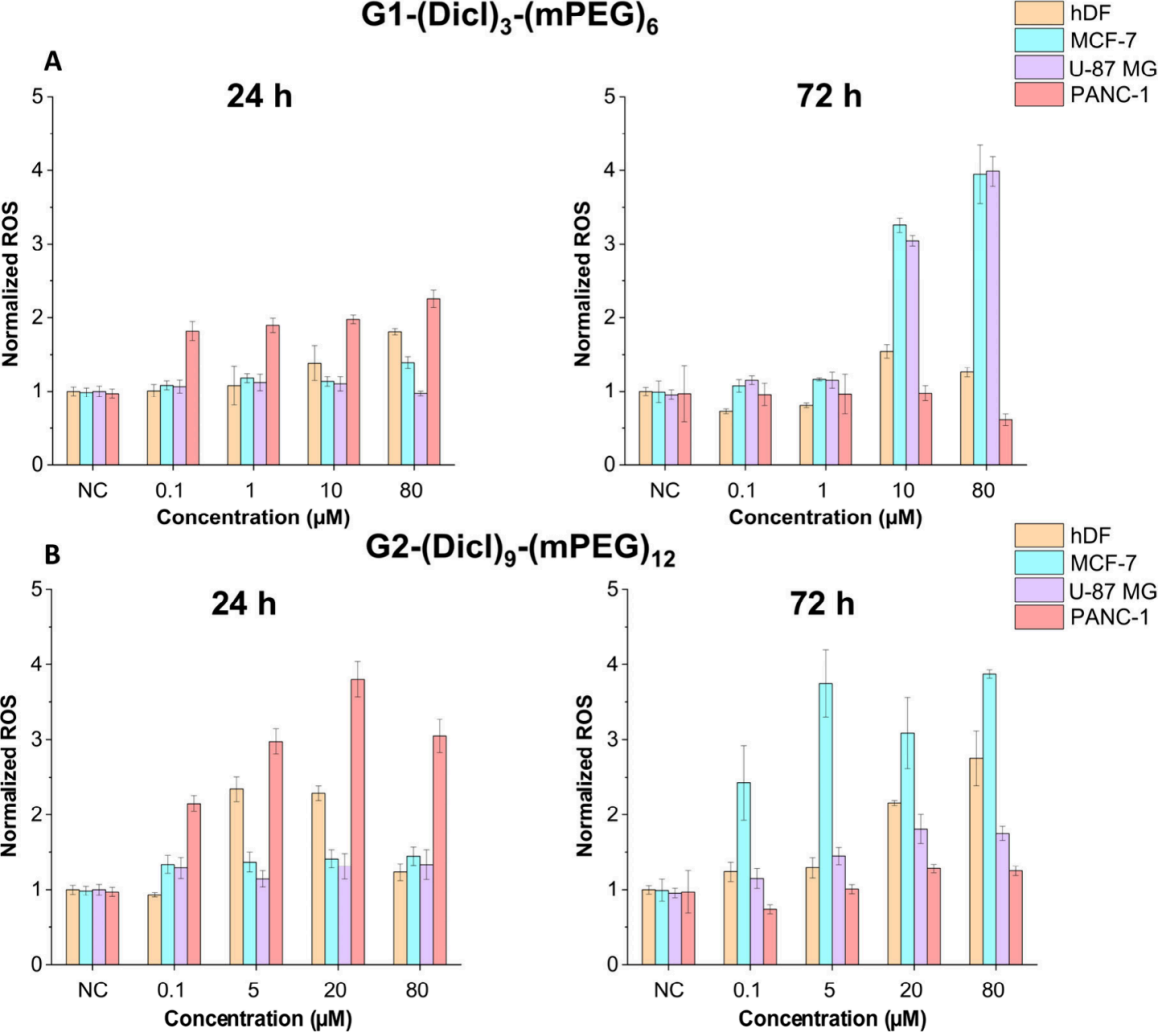
Time- and concentration-dependent ROS generation induced by (A) G1-(Dicl)_3_-(mPEG)_6_ and (B) G2-(Dicl)_9_-(mPEG)_12_ at 24 and 72 h across hDF, MCF-7, U-87 MG, and PANC-1 cell lines. Data was normalized to the percentage of viable cells obtained from the cytotoxicity assays and reported as mean ± SD (*n* = 2–3).

The ROS data strongly corroborated the time-dependent cytotoxicity patterns observed in the viability assays (Figure 5). At 24 h, most cell lines exhibited minimal ROS elevation (1–2 fold vs untreated control), consistent with the high cell viabilities (>70–90%) observed across all constructs at this time point. However, pronounced ROS induction emerged at 72 h, directly paralleling the cytotoxic effects and confirming oxidative stress as a major contributor to the anticancer response.

MCF-7 cells demonstrated the strongest ROS-cytotoxicity correlation, with both dendrimers generating substantial oxidative stress at concentrations corresponding to their cytotoxic activity. At 72 h, G1-(Dicl)_3_-(mPEG)_6_ induced robust ROS generation (3–4 fold) at 10 μM, while G2-(Dicl)_9_-(mPEG)_12_ reached comparable ROS levels at concentrations as low as 0.1 μM (Figure 5A,B). This direct correspondence between ROS generation and cytotoxic potency validates oxidative stress as an important mechanism contributing to the anticancer efficacy in MCF-7 cells.

U-87 MG cells exhibited pronounced ROS generation for the G1 dendrimer at concentrations corresponding to its cytotoxic range, reaching approximately 3-fold induction at 10 μM and 4-fold at 80 μM after 72 h treatment. In contrast, the G2 dendrimer produced substantially weaker ROS responses across all tested concentrations, directly mirroring its reduced cytotoxic efficacy in U-87 MG cells and supporting the generation-dependent selectivity observed in viability assays.

PANC-1 cells displayed a distinct ROS generation profile characterized by early oxidative stress induction that preceded cytotoxic effects. Both dendrimers, particularly G2-(Dicl)_9_-(mPEG)_12_, induced measurable ROS elevation (2–3 fold) within 24 h, which was reduced to baseline levels by 72 h. This reduction likely reflects the activation of cellular antioxidant defenses to mitigate oxidative stress. However, significant cytotoxicity became evident only after 72 h treatment, suggesting that prolonged oxidative challenge or partial exhaustion of protective mechanisms may be required to trigger cell death.

Normal hDF fibroblasts exhibited a moderate increase in ROS levels (2.5–3.0-fold) only at the highest tested concentration (80 μM) for the G2 dendrimer, while ROS levels for the G1 dendrimer remained near baseline. This response was observed primarily at 72 h and corresponded to the concentration range where pronounced cytotoxicity occurred for the G2 dendrimer and lower cell viability loss was recorded for the G1 construct. Critically, this indicates that cancer selectivity stems from differential cellular responses to oxidative stress rather than selective ROS generation. Cancer cells, with their altered metabolic profiles and compromised antioxidant defenses, are inherently more vulnerable to ROS-induced cell death compared to normal cells.^41^

Comparative analysis with free drug controls confirmed that oxidative stress generation is an inherent property of diclofenac,^8,42^ with both Dicl-Na and Dicl-mPEG capable of elevating ROS in cancer cells while sparing hDF fibroblasts (Figure S19). The dendritic constructs amplified this effect by generating controlled and sustained ROS at therapeutically relevant concentrations, achieving equivalent or greater oxidative stress at significantly lower doses than required for the free drug (Figure 5, Figure S20).

The time-dependent correlation between ROS induction and cytotoxicity confirmed that extended exposure (72 h) was required for effective drug release and oxidative stress accumulation. The delayed onset of cytotoxic effects, particularly in PANC-1 cells where early ROS induction preceded cell death, supports the controlled-release mechanism of the dendritic constructs and distinguishes them from immediate-release free drug formulations.^12^

In conclusion, ROS analysis provides mechanistic insight into an important pathway underlying the cytotoxicity profiles, demonstrating that oxidative stress generation contributes significantly to the observed anticancer activity. While diclofenac is known to exert anticancer effects through multiple mechanisms, the generation-dependent ROS patterns closely parallel the structure–activity relationships observed in cell viability assays, with G1-(Dicl)_3_-(mPEG)_6_ demonstrating consistent ROS generation across multiple cancer cell types while G2-(Dicl)_9_-(mPEG)_12_ showing more variable responses. These findings confirm that the diclofenac-PEGylated dendritic constructs effectively harness ROS-mediated anticancer effects within a controlled delivery framework, establishing a mechanistically informed and cancer-selective nanotherapeutic approach.

## Conclusions

This study demonstrates that rational dendritic engineering enables the repurposing of diclofenac as a promising scaffold for selective anticancer applications. AB_2_C-type HFDs enabled orthogonal conjugation of up to nine diclofenac moieties within the dendrimer interior while uniformly decorating the periphery with PEG chains, producing well-defined amphiphilic core–shell nanostructures that addressed diclofenac’s fundamental pharmacokinetic limitations while dramatically enhancing its anticancer potency.

The biological evaluation revealed clear structure–activity relationships that validate this dendritic design approach. G1-(Dicl)_3_-(mPEG)_6_ emerged as the most effective construct, demonstrating consistent anticancer activity across diverse cancer cell lines at low-micromolar concentrations while maintaining excellent safety profiles in noncancerous cells. The superior therapeutic performance of this lower-generation dendrimer, despite containing fewer drug molecules than G2-(Dicl)_9_-(mPEG)_12_, highlights that optimal nanotherapeutic design depends on balanced multivalent architecture rather than simply maximizing drug loading. The G2 HFD showed exceptional potency specifically in MCF-7 cells but reduced activity in other cancer models, suggesting its utility for the specified indication.

The mechanistic studies confirmed that ROS generation represents a key pathway contributing to the enhanced anticancer activity, with generation-dependent ROS patterns closely correlating with cytotoxicity profiles. Collectively, these findings establish design principles for tailoring dendritic scaffolds to specific oncological applications and provide a solid foundation for further preclinical investigation of diclofenac-PEGylated dendrimers.

## Supplementary Material



## Notes

Synthetic protocols of all diclofenac-PEGylated dendrimers and Dicl-mPEG linear conjugate, ^1^H and ^13^C NMR spectra (Figures S1, S2, S4–S6, S8) DOSY spectra (Figures S3, S7, S9), MALDI-TOF spectra (Figures S13–S15), SEC chromatographs (Figures S11, S12), FTIR spectra (Figure S10), DLS analysis (Figure S16), cytotoxicity assay (Figures S17, S18), and ROS generation (Figures S19, S20) (PDF)
