# Supplementary material for: PEGylated Heterofunctional Dendrimers Enable Multivalent Diclofenac Delivery for ROS-Driven Anticancer Activity
Source: ACS Appl Mater Interfaces. 2026 Feb 10;18(7):11134–44. doi: 10.1021/acsami.6c00115 (PMC12954661; doi:10.1021/acsami.6c00115)
Supplement: Supplementary file 1 [file am6c00115_si_001.pdf]

Supporting Information

PEGylated Heterofunctional Dendrimers Enable Multivalent Diclofenac Delivery for ROS-Driven Anticancer Activity

Arunika Singh[1], Natalia Sanz del Olmo[1,2,3] and Michael Malkoch[1]*

[1]Department of Fibre and Polymer Technology, KTH Royal Institute of Technology, Stockholm, 100 44, Sweden.

[2]Department of Organic and Inorganic Chemistry, Faculty of Sciences, Research Institute in Chemistry "Andrés M. Del Río" (IQAR), University of Alcala, Madrid, 28805, Spain.

[3]Institute "Ramón y Cajal" for Health Research (IRYCIS), Madrid, 28034, Spain.

*Author to whom correspondence should be addressed.

Corresponding author: Prof. Michael Malkoch

Contact info: School of Engineering Sciences in Chemistry, Biotechnology and Health; Department of Fibre and Polymer Technology; Division of Coating Technology Teknikringen 48, Stockholm, SE-10044, Sweden (malkoch@kth.se) Fax: (+) 46 (0)8 790 82 83.

Table of Contents

General information 4

Synthesis protocols 5

Figures 13

Figure S1. ^{1}H and ^{13}C NMR spectra of G1-(Dicl)$_3$-(OH)$_6$ in $(CD_3)_2CO$

Figure S2. ^{1}H and ^{13}C NMR spectra of G1-(Dicl)$_3$-(mPEG)$_6$ in $(CD_3)_2CO$

Figure S3. DOSY spectra of G1-(Dicl)$_3$-(mPEG)$_6$ in $(CD_3)_2CO$

Figure S4. ^{1}H and ^{13}C NMR spectra of G2-(Dicl)$_9$-(Ac)$_6$ in $CDCl_3$

Figure S5. ^{1}H and ^{13}C NMR spectra of G2-(Dicl)$_9$-(OH)$_{12}$ in $(CD_3)_2CO$

Figure S6. ^{1}H and ^{13}C NMR spectra of G2-(Dicl)$_9$-(mPEG)$_{12}$ in $(CD_3)_2CO$

Figure S7. DOSY spectra of G2-(Dicl)$_9$-(mPEG)$_{12}$ in $(CD_3)_2CO$

Figure S8. ^{1}H and ^{13}C NMR spectra of Dicl-mPEG in $(CD_3)_2CO$

Figure S9. DOSY spectra of Dicl-mPEG in $(CD_3)_2CO$

Figure S10. Stacked FTIR spectra of G2-(N$_3$)$_9$-(Ac)$_6$, G2-(Dicl)$_9$-(Ac)$_6$ and Dicl-alkyne

Figure S11. SEC of G2-(Dicl)$_9$-(Ac)$_6$

Figure S12. SEC overlay of G1-(Dicl)$_3$-(OH)$_6$ and G2-(Dicl)$_9$-(OH)$_{12}$

Figure S13. MALDI-TOF spectra of G2-(Dicl)$_9$-(OH)$_{12}$ in DCTB

Figure S14. MALDI-TOF spectra of G2-(Dicl)$_9$-(mPEG)$_{12}$ in DCTB

Figure S15. MALDI-TOF spectra of Dicl-mPEG in DHB

Figure S16. DLS analysis of Dicl-mPEG, G1-(Dicl)$_3$-(mPEG)$_6$ and G2-(Dicl)$_9$-(mPEG)$_{12}$ at 37 °C. Z-average size (nm, bars, left axis) and polydispersity indices (circles, right axis) at (A) 40 µM and (B) 500 µM. Summary of hydrodynamic diameters for representative constructs, including intensity- (D_i), volume- (D_v), and number-weighted (D[illegible]) diameters at (C) 40 µM and (D) 500 µM. Mean values accompanied by standard deviation (SD), n ≥ 3.

Figure S17. Cytotoxicity evaluation of Dicl-Na, Dicl-mPEG, G1-(Dicl)$_3$-(mPEG)$_6$ and G2-(Dicl)$_9$-(mPEG)$_{12}$ in hDF, U-87 MG, PANC-1 and MCF-7 cell lines after 24h treatment. Data are presented as mean ± SD (n = 3)

Figure S18. Cytotoxicity evaluation of Dicl-Na, Dicl-mPEG, G1-(Dicl)$_3$-(mPEG)$_6$, and G2-(Dicl)$_9$-(mPEG)$_{12}$ in hDF, MCF-7, U-87 MG, and PANC-1 cell lines after 72 hours of treatment at concentrations of 3 µM to 320 µM. Data are presented as mean ± SD (n = 3)

Figure S19. Time- and concentration-dependent ROS generation induced by (A) Dicl-Na and (B) Dicl-mPEG at 24 and 72 h across hDF, U-87 MG, PANC-1, and MCF-7 cell lines. Data was normalized to untreated controls and reported as mean ± SD (n = 2-3)

Figure S20. Time- and concentration-dependent ROS generation induced by (A) G1-(Dicl)$_3$-(mPEG)$_6$ (3-160 µM) and (B) G2-(Dicl)$_9$-(mPEG)$_{12}$ (160 µM) at 24 and 72 h across hDF, MCF-7, U-87 MG, and PANC-

1 cell lines. Data was normalized to the percentage of viable cells obtained from the cytotoxicity assays and reported as mean ± SD (n = 2-3)

References..**25**

General Information

Abbreviations

Ac	Acetonide
br	Broad signal
$CuSO_4.5H_2O$	Copper sulfate
DCC	N,N′-Dicyclohexylcarbodiimide
DCM	Dichloromethane
DCU	1,3-Dicyclohexyl urea
Dicl-alkyne	Diclofenac with terminal alkyne functionality
Dicl-Na	Diclofenac sodium salt
Dicl-mPEG	Diclofenac conjugated with methoxypoly(ethylene glycol)
DLS	Dynamic light scattering
DTCB	Trans-2-[3-(4-tert-Butylphenyl)-2-methyl-2-propenylidene]malononitrile
DHB	2,5-dihydroxybenzoic acid
DMAP	4-(Dimethylamino)pyridine
DMF	Dimethylformamide
EtOAc	Ethyl acetate
MALDI-TOF	Matrix-assisted laser desorption ionization time-of-flight
$mPEG_{11}$-OH	Methoxy poly(ethylene glycol)$_{11}$-alcohol
$mPEG_{11}$-PA	Methoxy poly(ethylene glycol)$_{11}$-propionic acid
Na ascorbate	Sodium ascorbate
NMR	Nuclear magnetic resonance
ROS	Reactive oxygen species
SEC	Size Exclusion Chromatography
$NaHCO_3$	Sodium bicarbonate
$NaHSO_4$	Sodium bisulfate
THF	Tetrahydrofuran

Synthesis protocols

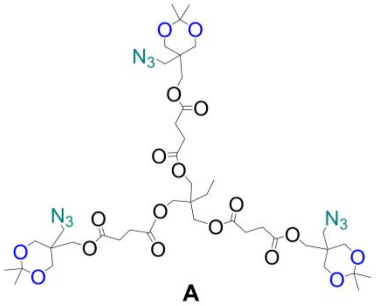

A

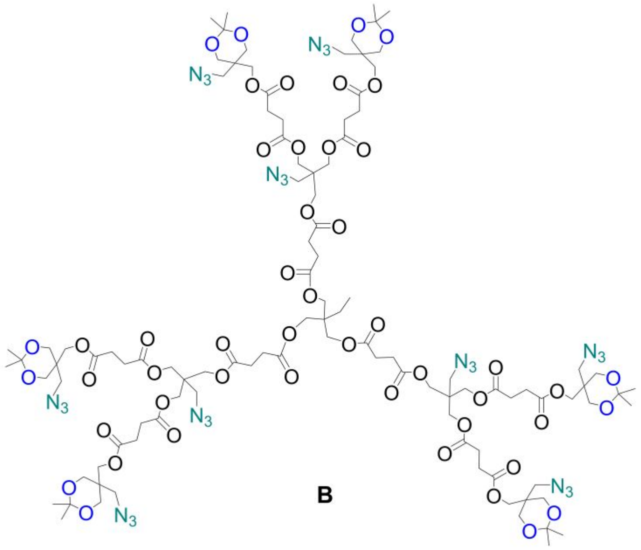

B

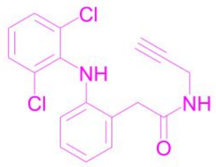

C

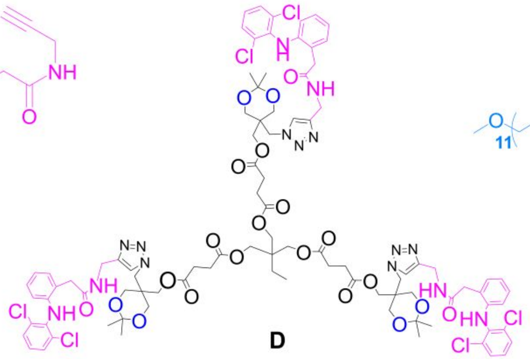

D

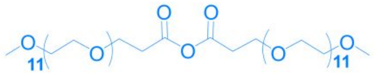

E

The compounds stated above were synthesized following previously reported procedures.[1,2] A) G1-$(N_3)_3$-$(Ac)_3$[1] B) G2-$(N_3)_9$-$(Ac)_6$[1] C) Dicl-alkyne[1] D) G1-$(Dicl)_3$-$(Ac)_3$[1] E) mPEG anhydride[1,2]

General procedure for post-functionalization of dendrimers with Dicl-alkyne

The post-functionalization of azide-terminated dendrimers[1] with Dicl-alkyne[1] was accomplished using CuAAC reaction approach adapted from a previously reported protocol[1], with modified purification steps. Briefly, the acetonide-protected azide dendrimer (1 eq) was stirred overnight at room temperature with Dicl-alkyne (1.5 eq per N_3 group) in a 1:1 mixture of THF and water, in the presence of $CuSO_4 \cdot 5H_2O$ and sodium ascorbate. Upon reaction completion, the crude product was washed 3 times with 0.5% w/w aqueous EDTA solution to remove $CuSO_4.5H_2O$ and Na ascorbate. Subsequently, the unreacted Dicl-alkyne was purified by passage through a silica plug, eluting initially with EtOAc:Hep (50:50) and concluding with EtOAc:MeOH (90:10) to yield the pure product.

General procedure for acetonide deprotection in the synthesis of hydroxyl-functional dendrimers

The Dicl functionalized acetonide-protected dendrimer was dissolved in MeOH, followed by addition of 12 wt% p-toluenesulfonic acid monohydrate. The reaction mixture was stirred at room temperature for few hours, and the reaction progress was monitored by NMR and MALDI-TOF. Upon completion, the acid was neutralized with an equimolar amount of pyridine to form pyridinium p-toluenesulfonate (PPTS). The solvent was evaporated, and the residue was dissolved in DCM and washed sequentially with aqueous $NaHCO_3$ (1×) and brine solution (2×) to remove residual p-TSA and PPTS. The organic layer was concentrated under vacuum to yield hydroxyl-functionalized dendrimers.

General procedure for anhydride-based esterification of hydroxyl-functional dendrimers

The esterification of dendritic peripheral hydroxyl groups with $mPEG_{11}$-PA was conducted following the previously reported anhydride-based esterification protocol with slight modifications.[1,2] Briefly, $mPEG_{11}$-PA (2 eq) was dissolved in a flask containing DCM and placed on an ice bath. A solution of DCC (1 eq) in DCM was then added dropwise over the cold reaction mixture and the reaction mixture was left to stir overnight. The resulting DCU precipitate formed in the reaction was removed by filtration through celite on the next day to obtain the mPEG anhydride[1,2]. Next, the hydroxyl functional dendrimer (1 eq) was dissolved in a flask containing DCM, to which the bases DMAP (0.2 eq/OH group) and pyridine (5 eq/OH group) were added. Subsequently, a solution of the freshly prepared mPEG anhydride[1,2] (1.6 eq/OH group) in DCM was introduced to the reaction mixture containing the precursor with bases and the reaction was stirred at room temperature overnight. The reaction completion was monitored through NMR spectroscopy. The crude product was concentrated in DCM and precipitated thrice in cold diethyl ether, followed by solvent removal to yield the PEGylated dendrimer.

General procedure for one-pot esterification of diclofenac with $mPEG_{11}$-OH

The esterification of diclofenac with $mPEG_{11}$-OH was performed using a one-pot, anhydride-based coupling approach. Diclofenac (2.0 eq) was dissolved in DCM and cooled on an ice bath. A solution of DCC (2.0 eq) in DCM was added dropwise, and the mixture was stirred at 0 °C for 2 h to form the diclofenac anhydride intermediate in situ. $mPEG_{11}$-OH (1.0 eq), pre-dried with toluene, was then added together with DMAP (2.0 eq) and pyridine (5.0 eq) without removing the DCU byproduct. The reaction was stirred at room temperature for 16 h, and completion was confirmed by NMR spectroscopy and MALDI-TOF. The crude mixture was concentrated and purified by passage through a silica plug, eluting first with 20:80 EtOAc:Hep, followed by 90:10 EtOAc:MeOH to yield the pure Dicl-mPEG conjugate.

G1-(Dicl)$_3$-(OH)$_6$

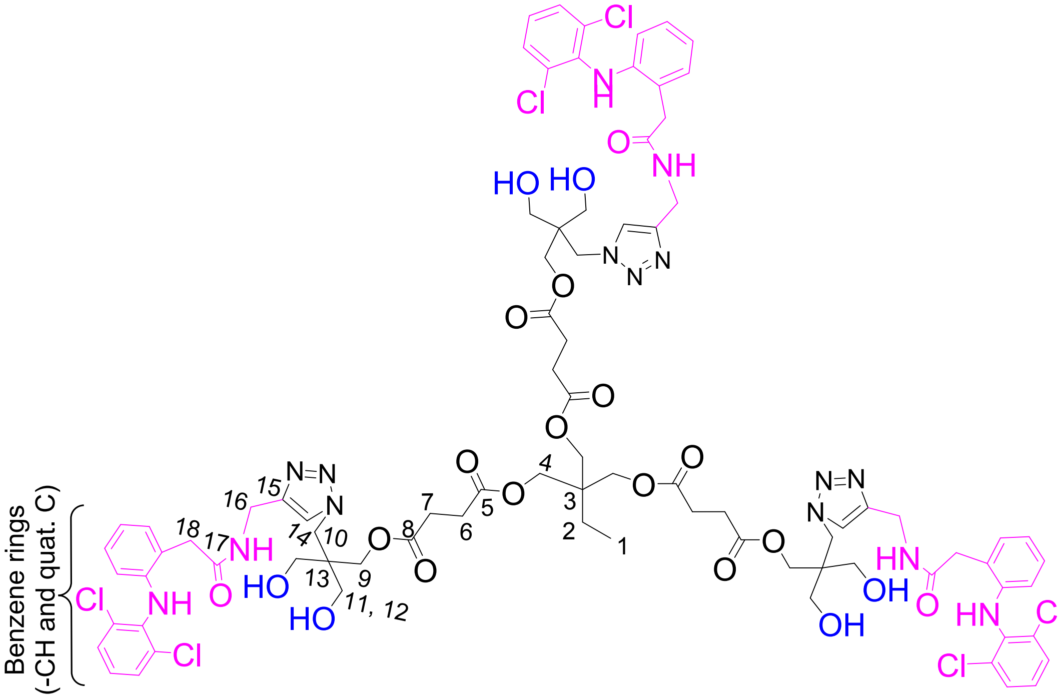

G1-(Dicl)$_3$-(OH)$_6$ was synthesized following the general procedure for acetonide deprotection, using the following reagents in the specified amounts: G1-(Dicl)$_3$-(Ac)$_3$[1] (98.6 mg, 0.050 mmol) and p-TSA (11.8 mg, 0.062 mmol). G1-(Dicl)$_3$-(OH)$_6$ was obtained as a viscous oil (90.0 mg, 97%). $C_{84}H_{95}Cl_6N_{15}O_{21}$ (1863.47 g mol^{-1}). ^{1}H-NMR (400 MHz, $(CD_3)_2CO$) δ/ppm: 8.41 (3H, s, br: NH), 8.18 (3H, m, br: NH), 7.87 (3H, s, H14), 7.43 (6H, m, -C*H* benzene rings), 7.23 (3H, m, -C*H* benzene rings), 7.14 – 6.98 (6H, m, -C*H* benzene rings), 6.84 (3H, m, -C*H* benzene rings), 6.42 (3H, m, -C*H* benzene rings), 4.49 (12H, m, H10, H18), 4.16 (6H, m, br: OH), 4.04 (12H, m, H4, H9), 3.76 (6H, s, H16), 3.50 (12H, m, H11, H12), 2.61 (12H, m, H6, H7), 1.48 (3H, m, H2), 0.88 (9H, m, H1). ^{13}C-NMR (101 MHz, $(CD_3)_2CO$) δ/ppm: 172.92 (C5, C8, C17), 172.72 (C5, C8, C17), 172.70 (C5, C8, C17), 144.32 (C15), 138.80, 131.39, 130.49, 129.84, 128.17, 126.67, 125.45 (-CH and quat. C benzene rings), 125.22 (C14), 121.90, 117.69 (-CH and quat. C benzene rings), 64.69 (C4), 64.15 (C9), 61.62 (C11, C12), 50.15 (C10), 45.97 (C13), 41.65 (C3), 40.80 (C16), 35.66 (C18), 29.55 (C6, C7), 23.60 (C2), 7.71 (C1). MALDI: Calc. [M+Na$^+$] = 1886.46 Da, Found [M+Na$^+$] = 1886.26 Da. SEC (DMF) M_n = 2345.6 g mol^{-1}, M_w= 2426.3 g mol^{-1}, Đ = 1.03.

G1-(Dicl)$_3$-(mPEG)$_6$

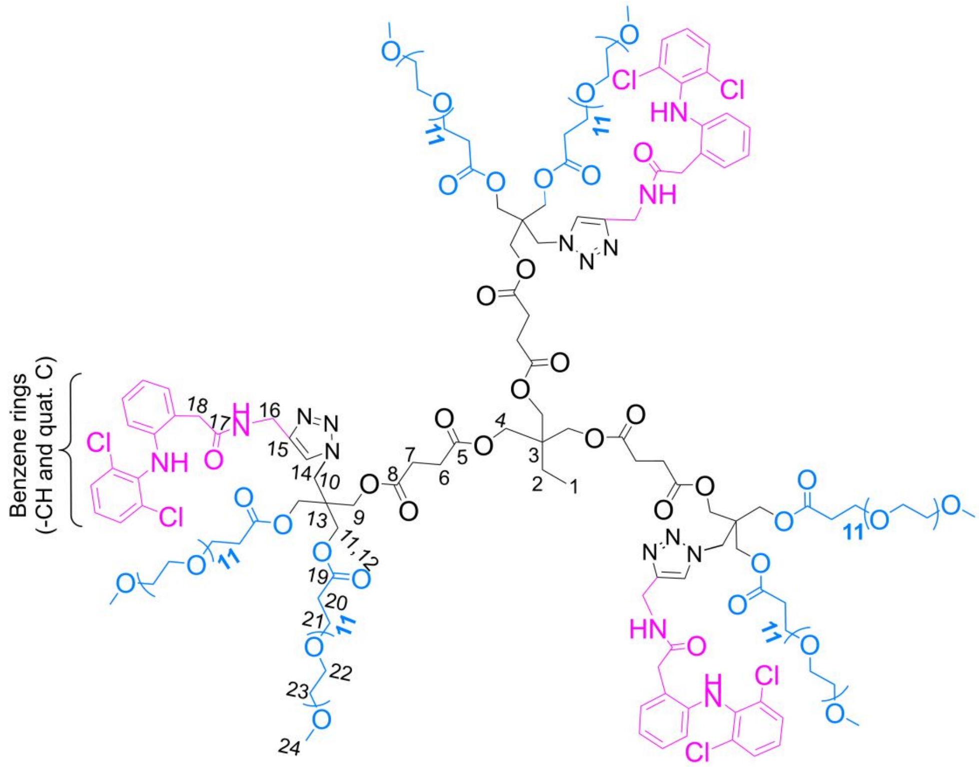

G1-(Dicl)$_3$-(OH)$_6$ (72.0 mg, 0.039 mmol) underwent esterification with mPEG anhydride (403.2 mg, 0.35 mmol) following the general esterification procedure. The reaction was carried out in DCM (3 mL) with DMAP (14.0 mg, 0.12 mmol) and pyridine (300 µL, 0.37 mmol). G1-(Dicl)$_3$-(mPEG)$_6$ was freeze-dried and obtained as a colorless oil (136 mg, 66%). $C_{240}H_{395}Cl_6N_{15}O_{99}$ (5287.51 g mol^{-1}). ^{1}H-NMR (400 MHz, $(CD_3)_2CO$) δ/ppm: 8.56 (3H, s, br: NH), 8.16 (3H, s, br: NH), 7.91 (3H, s, H14), 7.46 (6H, m, -C*H* benzene rings), 7.29 – 7.21 (3H, m, -C*H* benzene rings), 7.17 – 7.01 (6H, m, -C*H* benzene rings), 6.91 – 6.80 (3H, m, -C*H* benzene rings), 6.43 (3H, m, -C*H* benzene rings), 4.63 (6H, m, H10, H18), 4.49 (6H, s, H10, H18), 4.21 – 4.03 (24H, m, H4, H9, H11, H12), 3.79 – 3.42 (296H, m, H16, H20, H22, H23), 3.28 (18H, s, H24), 2.69 – 2.57 (24H, m, H6, H7, H21), 1.51 (2H, m, H2), 0.88 (3H, m, H1). ^{13}C-NMR (101 MHz, $(CD_3)_2CO$) δ/ppm: 172.86 (C5, C8, C17), 172.61 (C5, C8, C17), 172.37 (C5, C8, C17), 171.45 (C19), 145.45 (-CH and quat. C benzene rings), 144.38 (C15), 138.88, 131.47, 130.42, 129.90, 128.13, 126.86, 125.57 (-CH and quat. C benzene rings), 125.22 (C14), 121.89, 117.70 (-CH and quat. C benzene rings), 72.60 (C22, C23), 71.18 (C22, C23), 71.12 (C22, C23), 71.07 (C22, C23), 71.00 (C22, C23), 67.18 (C20), 64.73 (C4, C9), 63.72 (C4, C9), 63.27 (C11, C12), 58.79 (C24), 50.45 (C10), 43.70 (C13), 41.67 (C3), 40.85 (C16), 35.70 (C18, C21), 35.62 (C18, C21), 29.54 (C6, C7), 23.68 (C2), 7.81 (C1). MALDI: Calc. [M+Na$^+$] = 5310.49 Da, Found [M+Na$^+$] = 5311.18 Da. SEC (DMF) M_n = 5803.8 g mol^{-1}, M_w= 5974.8 g mol^{-1}, Đ = 1.02.

G2-(Dicl)$_9$-(Ac)$_6$

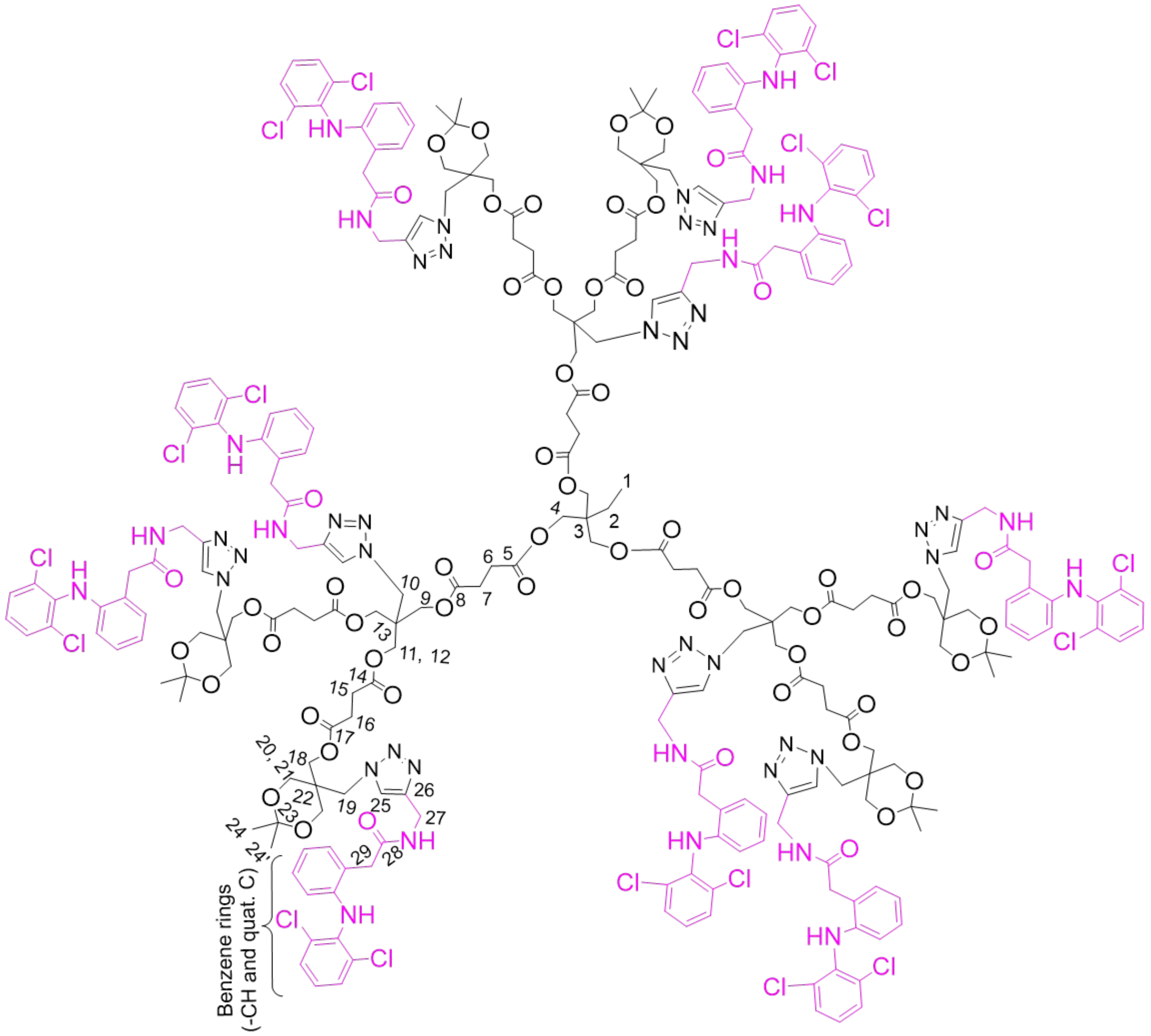

G2-(N_3)$_9$-(Ac)$_6$ (65.0 mg, 0.025 mmol) was post-functionalized with Dicl-alkyne (114 mg, 0.34 mmol) in 4 mL of THF:H_2O (1:1) following the general CuAAC procedure. The reaction was catalyzed by $CuSO_4 \cdot 5H_2O$ (11.4 mg, 0.045 mmol) in the presence of sodium ascorbate (18.0 mg, 0.091 mmol). G2-(Dicl)$_9$-(Ac)$_6$ was obtained as a colourless oil (120.0 mg, 86%). $C_{258}H_{281}Cl_{18}N_{45}O_{57}$ (5562.44 g mol^{-1}).^{1}H-NMR (400 MHz, $CDCl_3$) δ/ppm: 7.66 (27H, m, H25, br: NH), 7.29 (18H ,m, -C*H* benzene rings), 7.12 (9H, m, -C*H* benzene rings), 7.02 (9H, m, -C*H* benzene rings), 6.93 (9H, m, -C*H* benzene rings), 6.81 (9H, m, -C*H* benzene rings), 6.45 (9H, m, -C*H* benzene rings), 4.48 (36H, m, H10, H19, H29), 4.06 – 3.98 (24H, m, H4, H9, H18), 3.87 (12H, m, H11, H12), 3.68 (30H, m, H20, H21, H27), 3.53 (12H, m, H20, H21, H27), 2.56 (36H, s, H6, H7, H15, H16), 1.38 (38H, m, H2, H24, H24'), 0.82 (3H, m, H1). ^{13}C-NMR (101 MHz, $CDCl_3$) δ/ppm: 172.20 (C5, C8, C14, C17, C28), 172.13 (C5, C8, C14, C17, C28), 172.06 (C5, C8, C14, C17, C28), 171.91 (C5, C8, C14, C17, C28), 171.75 (C5, C8, C14, C17, C28), 171.71 (C5, C8, C14, C17, C28), 144.51 (-CH and quat. C benzene rings), 143.11 (C26), 143.09 (C26), 137.82, 137.80, 130.78, 129.96, 129.93, 128.91, 127.78, 125.05 (-CH and quat. C benzene rings), 124.97 (C25), 124.13, 121.42, 117.42 (-CH and quat. C benzene rings), 98.92 (C23), 64.23 (C4, C9, C11, C12, C18, C20, C21), 63.69 (C4, C9, C11, C12, C18, C20, C21), 62.88 (C4, C9, C11, C12, C18, C20, C21), 50.01 (C10, C19), 42.83 (C13), 40.81 (C3), 40.58 (C27), 38.06 (C22), 34.98 (C29), 28.83 (C6, C7, C15, C16), 25.55 (C24, C24'), 21.87 (C24,

C24’), 7.45 (C1). MALDI: Calc. [M+Na$^+$] = 5585.43 Da, Found [M+Na$^+$] = 5586.73 Da. SEC (DMF) M_n = 6134.4 g mol^{-1}, M_w= 6321.5 g mol^{-1}, Đ = 1.03.

G2-(Dicl)$_9$-(OH)$_{12}$

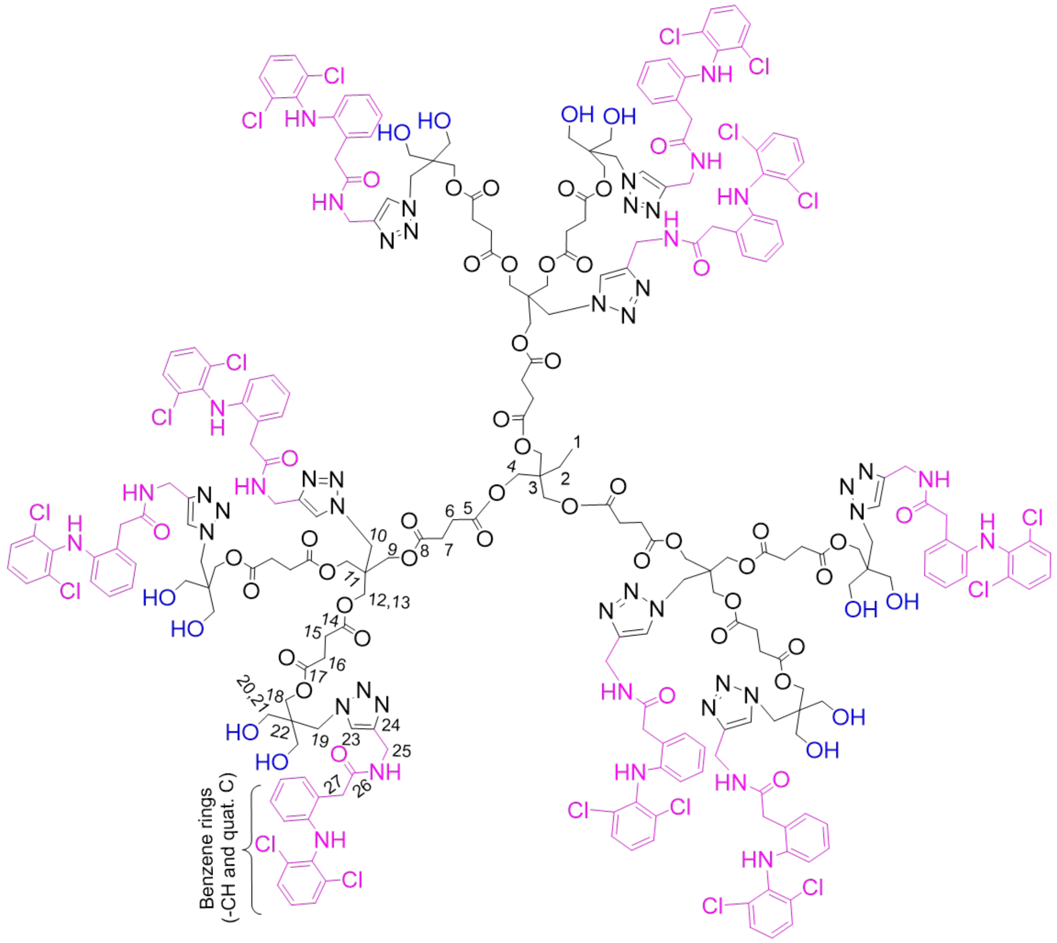

G2-(Dicl)$_9$-(OH)$_{12}$ was synthesized following the general procedure for acetonide deprotection, using the following reagents in the specified amounts: G2-(Dicl)$_9$-(Ac)$_6$ (101.4 mg, 0.018 mmol) and p-TSA (12.2 mg, 0.062 mmol). G2-(Dicl)$_9$-(OH)$_{12}$ was obtained as a viscous oil (50.0 mg, 52%). $C_{240}H_{257}Cl_{18}N_{45}O_{57}$ (5322.05 g mol^{-1}). ^{1}H-NMR (400 MHz, $(CD_3)_2CO$) δ/ppm: 8.42 – 8.17 (18H, m, br: NH), 7.94 – 7.83 (9H, m, H23), 7.41 (18H, m, -C*H* benzene rings), 7.21 (9H, m, -C*H* benzene rings), 7.11 – 6.98 (18H, m, -C*H* benzene rings), 6.86 – 6.78 (9H, m, -C*H* benzene rings), 6.40 (9H, m, -C*H* benzene rings), 4.65 – 4.42 (36H, m, H10, H19, H27), 4.25 – 3.99 (46H, m, H4, H9, H12, H13, br: OH), 3.75 (18H, s, H25), 3.55 – 3.42 (24H, m, H20, H21), 2.61 (36H, m, H6, H7, H15, H16), 0.91 – 0.82 (3H, m, H1). ^{13}C-NMR (101 MHz, $(CD_3)_2CO$) δ/ppm: 173.11 (C5, C8, C14, C17, C26), 173.00 (C5, C8, C14, C17, C26), 172.87 (C5, C8, C14, C17, C26), 172.82 (C5, C8, C14, C17, C26), 172.59 (C5, C8, C14, C17, C26), 145.43, 145.19 (-CH and quat. C benzene rings), 144.34 (C24), 144.31 (C24), 138.82, 138.79, 131.51, 131.46, 130.54, 130.52, 129.88, 128.24, 128.21, 126.70, 126.64, 125.68, 125.52 (-CH and quat. C benzene rings), 125.30 (C23), 125.27 (C23), 121.99, 121.96, 121.89, 117.72 (-CH and quat. C benzene rings), 64.86 (C4, C9, C12, C13, C18, C20, C21), 64.27 (C4, C9, C12, C13, C18, C20, C21), 63.71 (C4, C9, C12, C13, C18, C20, C21), 63.31 (C4, C9, C12, C13, C18, C20, C21), 61.62 (C4, C9, C12, C13, C18, C20, C21), 50.59 (C10, C19), 50.19 (C10, C19), 46.01 (C22), 43.59 (C11), 41.67 (C3, C25), 40.83 (C3, C25), 35.71 (C27), 28.64 (C6, C7, C15, C16), 23.30 (C2), 7.85 (C1). MALDI: Calc. [M+H$^+$] = 5323.06 Da, Found [M+H$^+$] = 5323.1 Da. SEC (DMF) M_n = 6073.6 g mol^{-1}, M_w= 6287.6 g mol^{-1}, Đ = 1.03.

G2-(Dicl)$_9$-(mPEG)$_{12}$

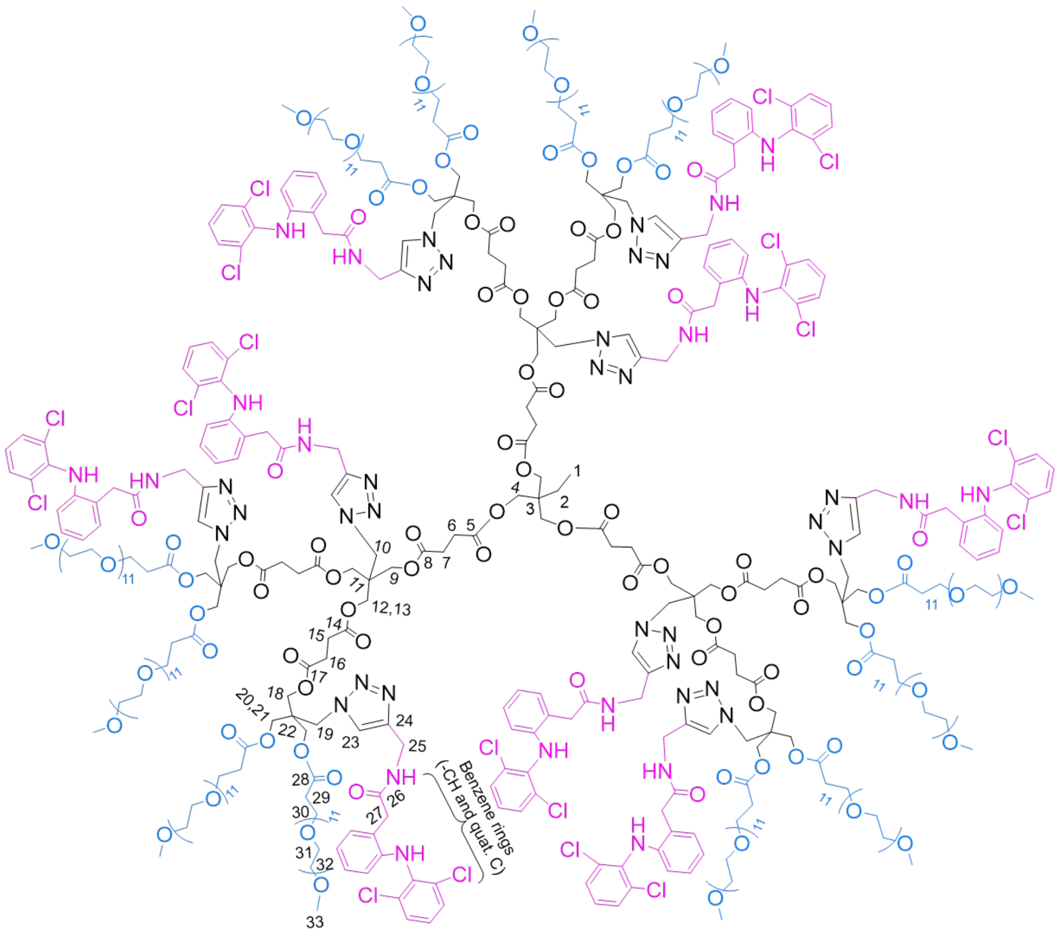

G2-(Dicl)$_9$-(OH)$_{12}$ (50.0 mg, 0.009 mmol) underwent esterification with mPEG anhydride (180.4 mg, 0.16 mmol) following the general esterification procedure. The reaction was carried out in DCM (3 mL) with DMAP (6.0 mg, 0.052 mmol) and pyridine (134 µL, 1.66 mmol). G2-(Dicl)$_9$-(mPEG)$_{12}$ was freeze-dried and obtained as a colorless oil (77 mg, 70%). $C_{552}H_{857}Cl_{18}N_{45}O_{213}$ (12170.13 g mol^{-1}). ^{1}H-NMR (400 MHz, $(CD_3)_2CO$) δ/ppm: 8.57 – 8.47 (9H, m, br: NH), 8.28 – 8.14 (9H, m, br: NH), 7.93 (9H, m, H23), 7.45 (18H, m, -C*H* benzene rings), 7.25 (9H, m, -C*H* benzene rings), 7.16 – 7.00 (18H, m, -C*H* benzene rings), 6.86 (9H, m, , -C*H* benzene rings), 6.42 (9H, m, -C*H* benzene rings), 4.67 – 4.45 (36H, m, H10, H19, H27), 4.23 – 4.03 (60H, m, H4, H9, H12, H13, H20, H21), 3.82 – 3.44 (570H, m, H25, H29, H31, H32), 3.28 (36H, s, H33), 2.72 – 2.55 (60H, m, H6, H7, H15, H16, H30), 1.48 (2H, m, H2), 0.87 (3H, m, H1). ^{13}C-NMR (101 MHz, $(CD_3)_2CO$) δ/ppm: 172.97 (C5, C8, C14, C17, C26, C28), 172.95 (C5, C8, C14, C17, C26, C28), 172.76 (C5, C8, C14, C17, C26, C28), 172.53 (C5, C8, C14, C17, C26, C28), 172.51 (C5, C8, C14, C17, C26, C28), 172.49 (C5, C8, C14, C17, C26, C28), 171.54 (C5, C8, C14, C17, C26, C28), 145.51 (-CH and quat. C benzene rings), 144.41 (C24), 144.38 (C24), 138.92, 138.89, 131.55, 130.49, 130.47, 129.95, 128.19, 126.94, 126.91, 126.88, 125.65 (-CH and quat. C benzene rings), 125.29 (C23), 125.26 (C23), 121.98, 121.96, 117.75 (-CH and quat. C benzene rings), 72.64 (C31, C32), 71.21 (C31, C32), 71.16 (C31, C32), 71.10 (C31, C32), 71.04 (C31, C32), 67.22 (C29), 64.81 (C4, C9, C12, C13, C18, C20, C21), 64.31 (C4, C9, C12, C13, C18, C20, C21), 63.85 (C4, C9, C12, C13, C18, C20, C21), 63.33 (C4, C9, C12, C13, C18, C20, C21), 58.83 (C33), 50.52 (C10, C19), 44.95 (C11), 43.75 (C22), 43.59 (C22), 41.72 (C3, C25), 40.89 (C3, C25), 40.07 (C3, C25), 35.75 (C27), 35.69 (C27), 27.70 (C6, C7, C15, C16), 23.72 (C2), 7.92 (C1). MALDI: Calc. $[M+K^+]$ = 12209.23 Da, Found $[M+K^+]$ = 12228.14 Da. SEC (DMF) M_n = 11112 g mol^{-1}, M_w= 11429 g mol^{-1}, Đ = 1.02.

Dicl-mPEG

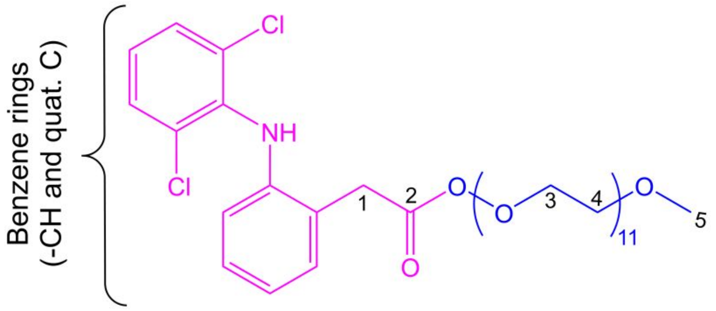

Diclofenac (125.9 mg, 0.42 mmol) was reacted with $mPEG_{11}$-OH (51 mg, 0.098 mmol) in DCM (2 mL) using DCC (44 mg, 0.25 mmol), DMAP (24.1 mg, 0.23 mmol), and pyridine (40 µL, 0.58 mmol) according to the general one-pot esterification procedure. Dicl-mPEG was obtained as a colorless oil (59 mg, 74%). $C_{37}H_{57}Cl_2NO_{14}$ (810.76 g mol^{-1}). ^{1}H-NMR (400 MHz, $(CD_3)_2CO$) δ/ppm: 7.40 (2H, s, -C*H* benzene rings), 7.22 (1H, m, -C*H* benzene rings), 7.11 – 6.99 (2H, m, -C*H* benzene rings), 6.82 (1H, m, -C*H* benzene rings), 6.40 (1H, m, -C*H* benzene rings), 3.63 – 3.45 (44H, m, H3, H4), 3.29 (3H, s, H5), 3.12 (2H, s, H1). ^{13}C-NMR (101 MHz, $(CD_3)_2CO$) δ/ppm: 177.22 (C2), 144.14, 144.00, 139.22, 131.56, 130.30, 129.93, 129.80, 127.79, 127.51, 124.84, 121.75, 118.33, 117.58 (-CH and quat. C benzene rings), 73.33 (C3, C4), 72.57 (C3, C4), 71.11 (C3, C4), 71.09 (C3, C4), 71.07 (C3, C4), 71.05 (C3, C4), 71.03 (C3, C4), 71.00 (C3, C4), 70.98 (C3, C4), 70.97 (C3, C4), 70.95 (C3, C4), 70.91 (C3, C4), 70.76, (C3, C4) 61.70 (C3, C4), 58.79 (C5), 39.59 (C1). MALDI: Calc. $[M+H^+]$ = 811.77 Da, Found $[M+H^+]$ = 812.20 Da. SEC (DMF) M_n = 763.78 g mol^{-1}, M_w= 778.75 g mol^{-1}, Đ = 1.01.

Figures

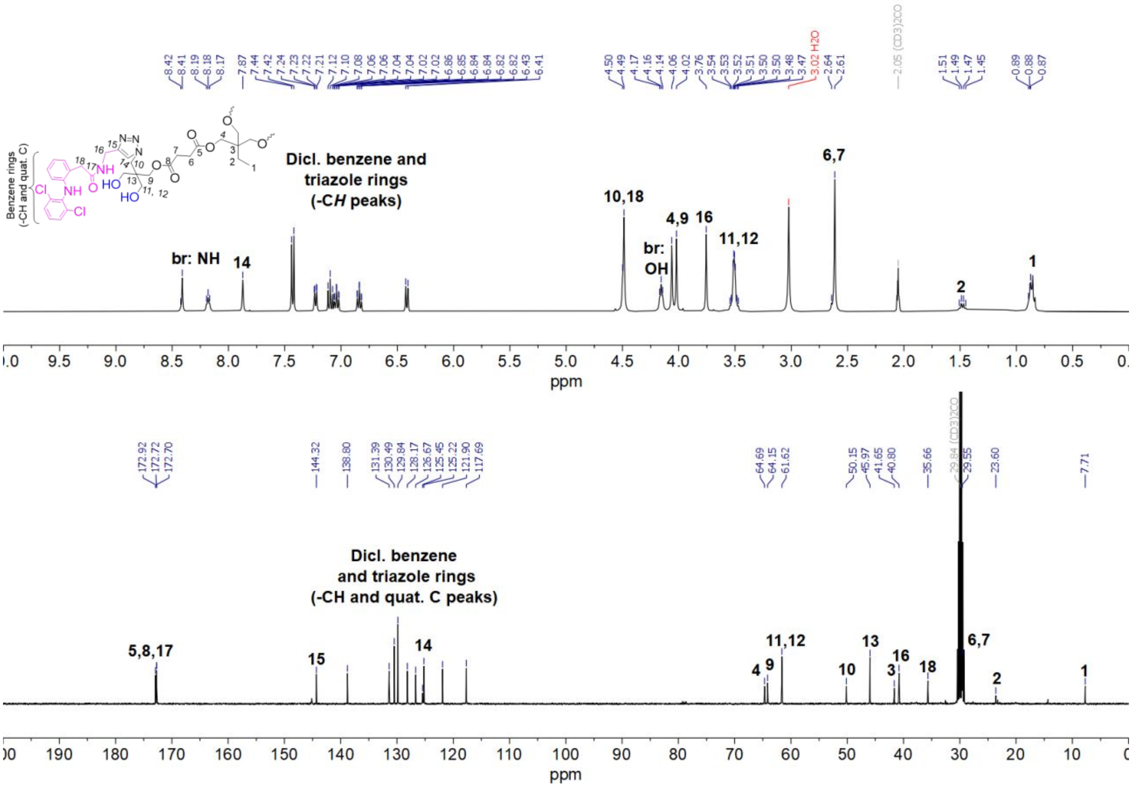

Figure S1. ^{1}H and ^{13}C NMR spectra of G1-(Dicl)$_3$-(OH)$_6$ in $(CD_3)_2CO$.

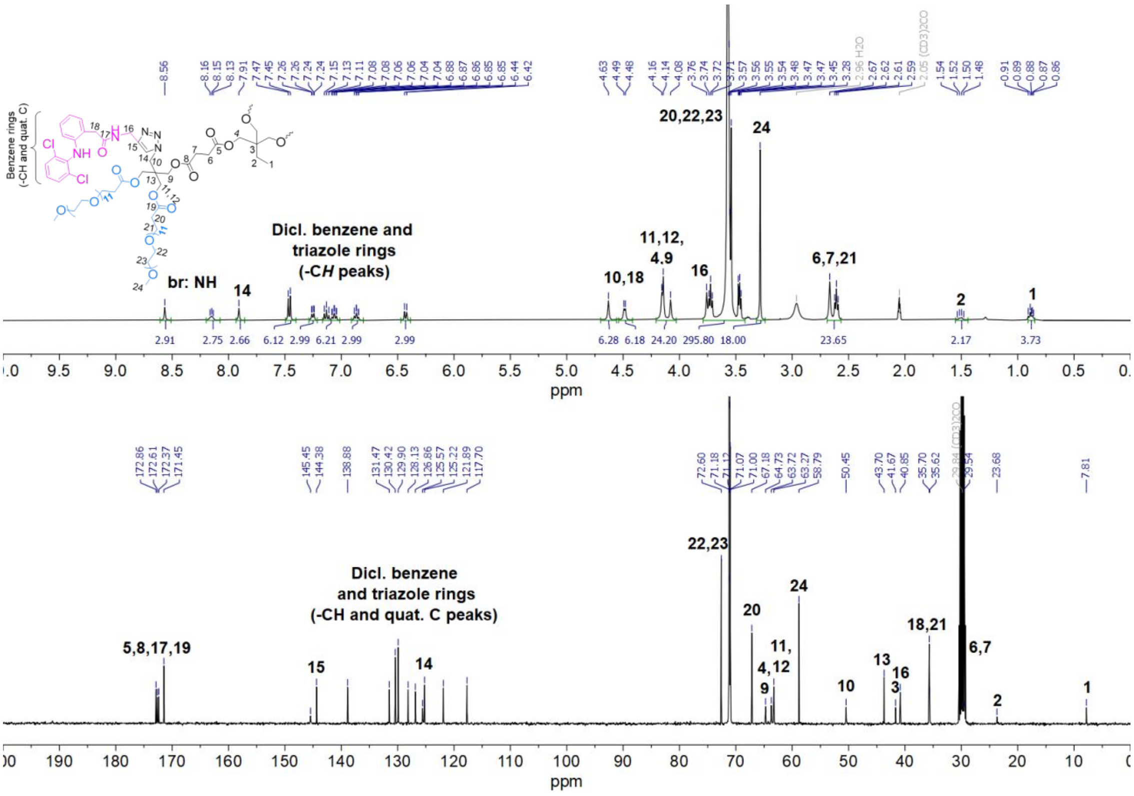

Figure S2. ^{1}H and ^{13}C NMR spectra of G1-(Dicl)$_3$-(mPEG)$_6$ in $(CD_3)_2CO$.

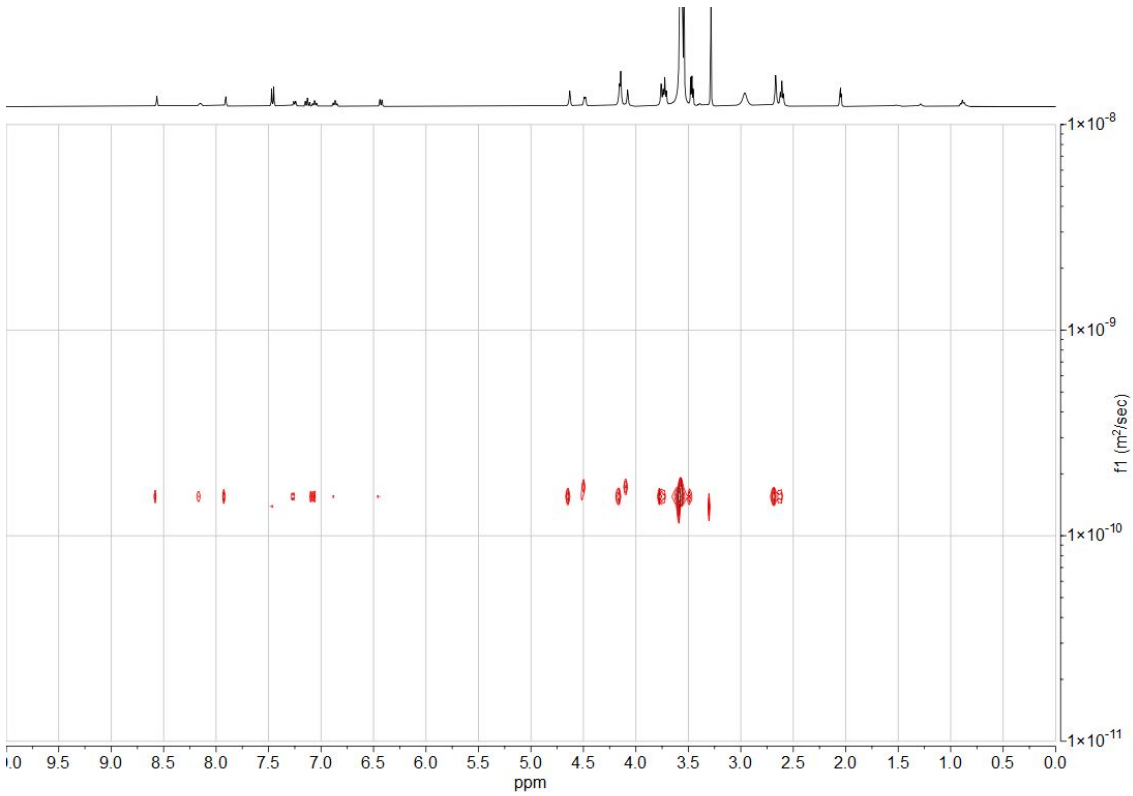

Figure S3. DOSY spectra of G1-$(Dicl)_3$-$(mPEG)_6$ in $(CD_3)_2CO$.

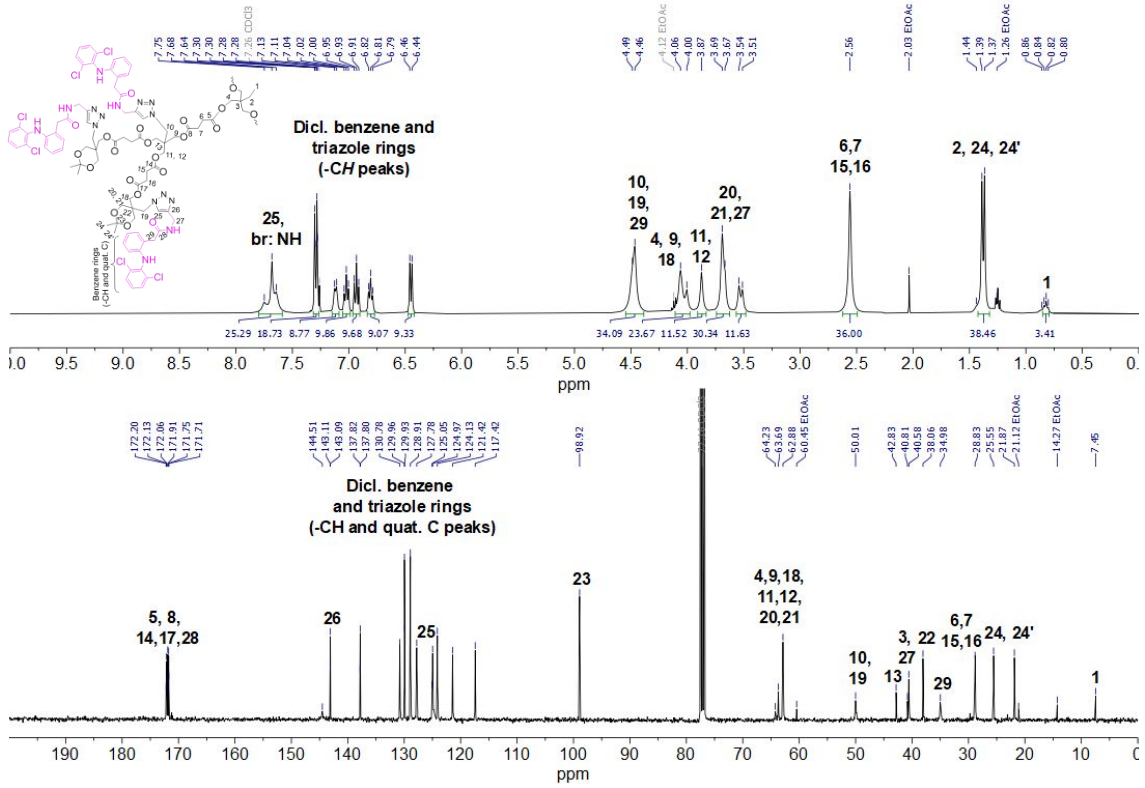

Figure S4. ^1H and ^{13}C NMR spectra of G2-$(Dicl)_9$-$(Ac)_6$ in $CDCl_3$.

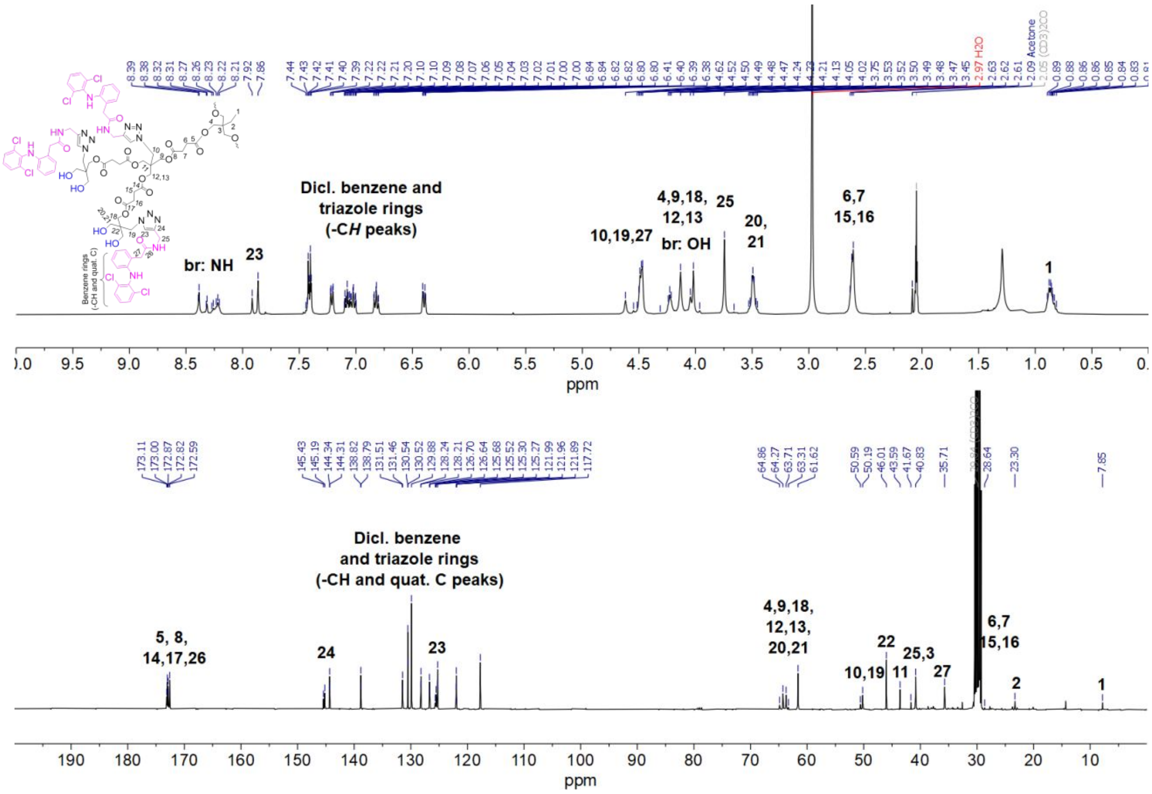

Figure S5. ^{1}H and ^{13}C NMR spectra of G2-(Dicl)$_9$-(OH)$_{12}$ in $(CD_3)_2CO$.

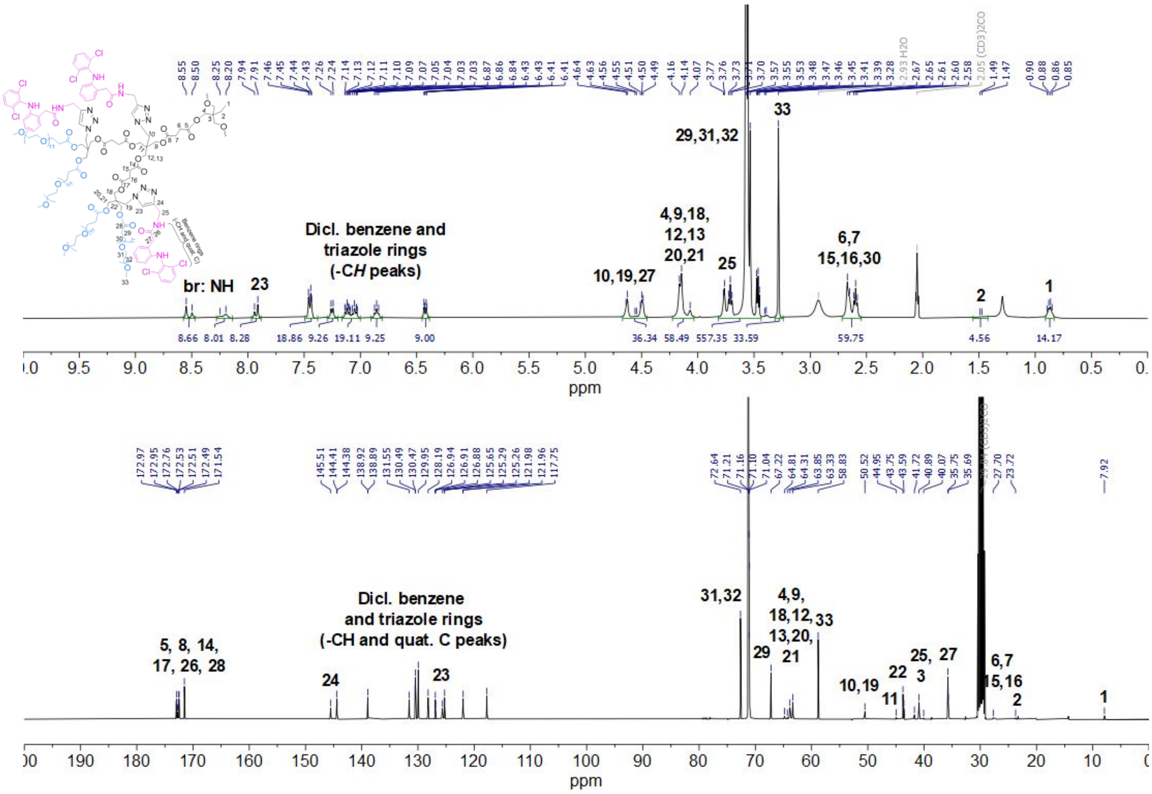

Figure S6. ^{1}H and ^{13}C NMR spectra of G2-(Dicl)$_9$-(mPEG)$_{12}$ in $(CD_3)_2CO$.

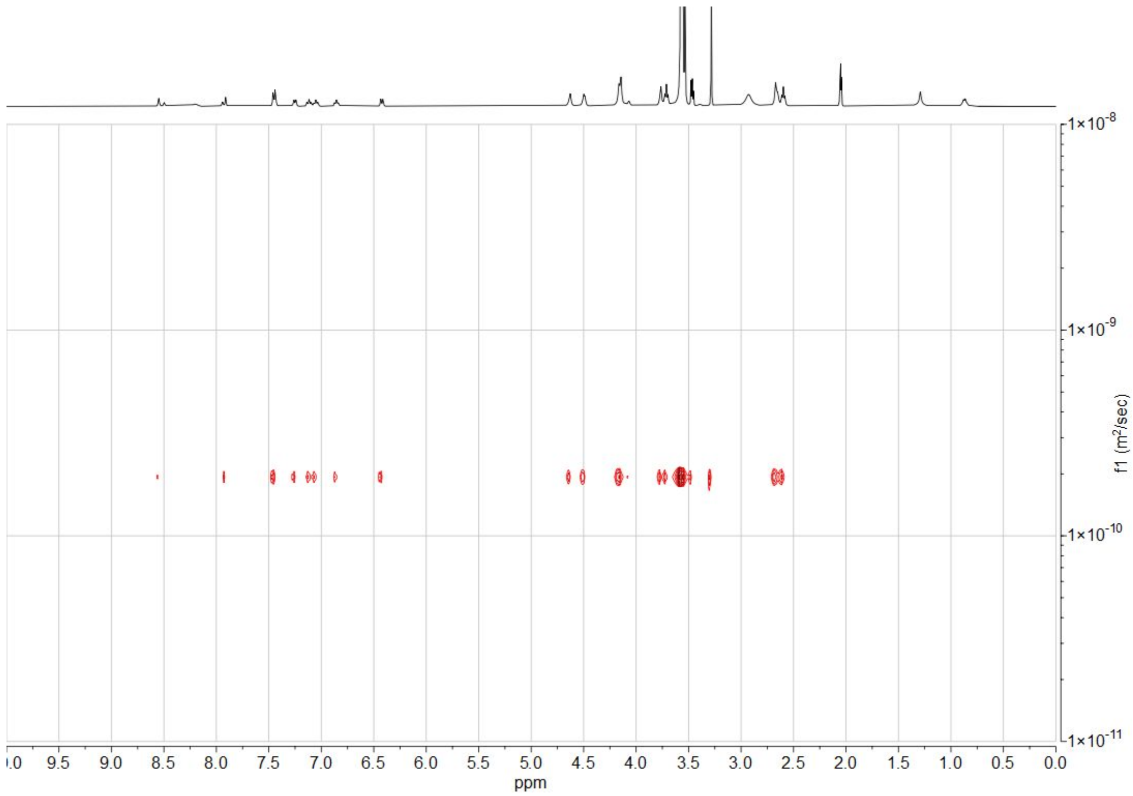

Figure S7. DOSY spectra of G2-$(Dicl)_9$-$(mPEG)_{12}$ in $(CD_3)_2CO$.

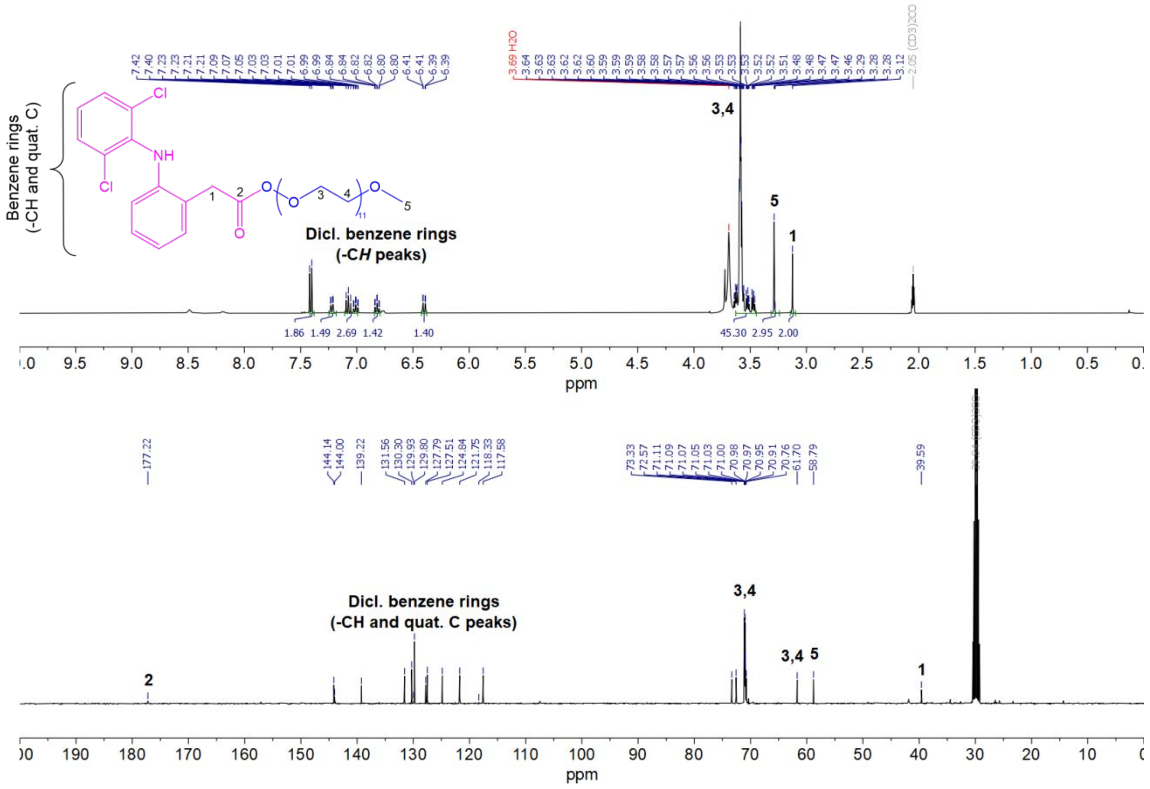

Figure S8. ^{1}H and ^{13}C NMR spectra of Dicl-mPEG in $(CD_3)_2CO$.

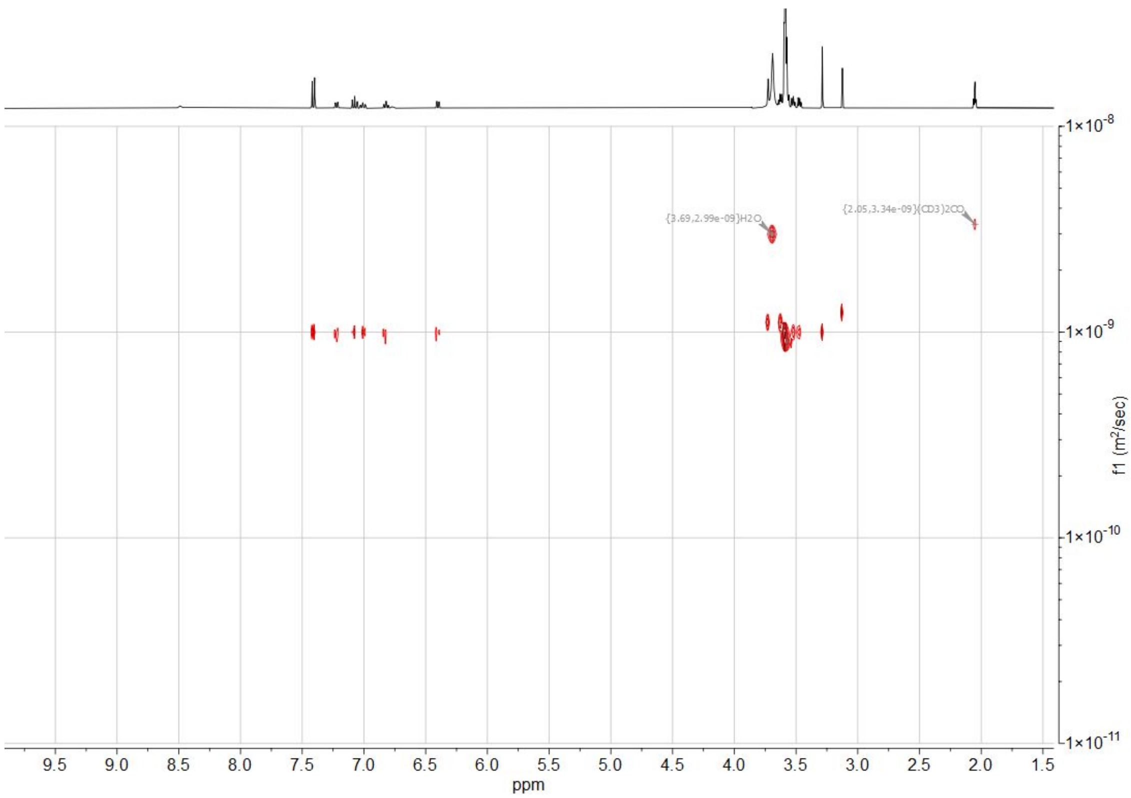

Figure S9. DOSY spectra of Dicl-mPEG in $(CD_3)_2CO$.

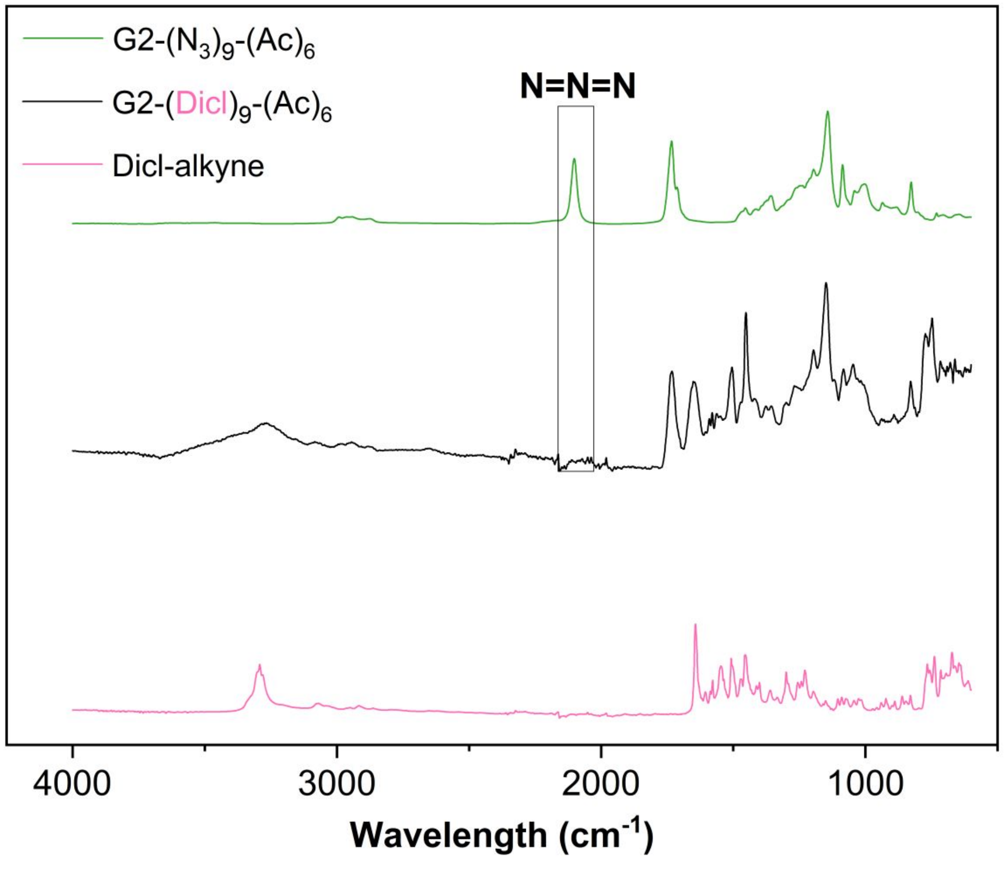

Figure S10. Stacked FTIR spectra of G2-$(N_3)_9$-$(Ac)_6$, G2-$(Dicl)_9$-$(Ac)_6$ and Dicl-alkyne.

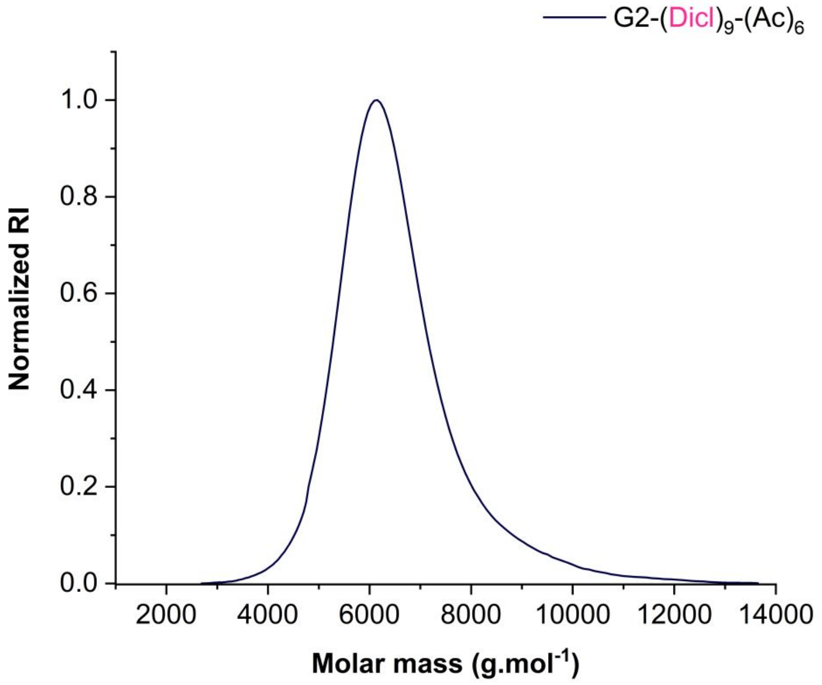

Figure S11. SEC of G2-$(Dicl)_9$-$(Ac)_6$.

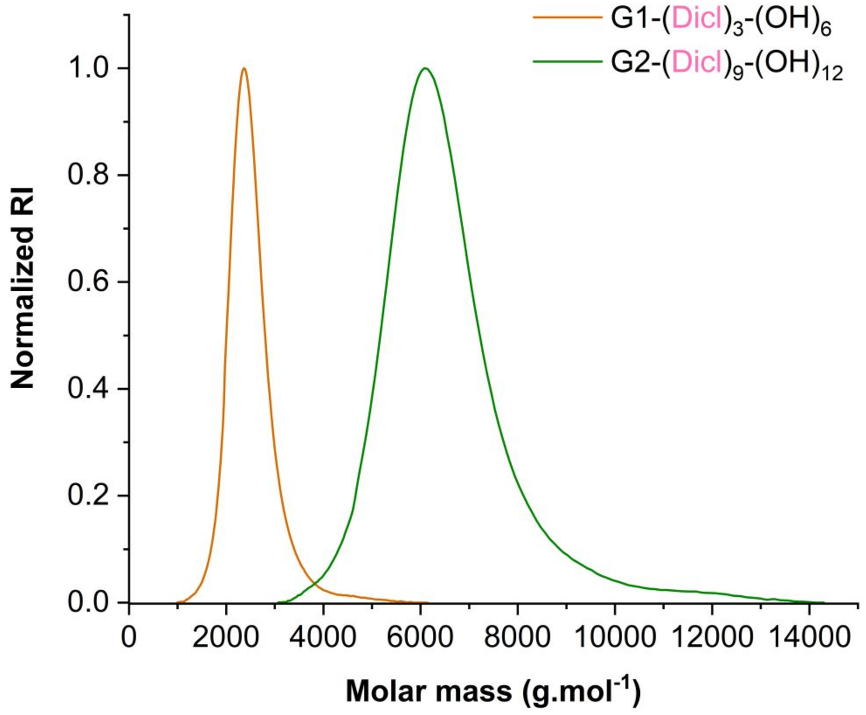

Figure S12. SEC overlay of G1-$(Dicl)_3$-$(OH)_6$ and G2-$(Dicl)_9$-$(OH)_{12}$.

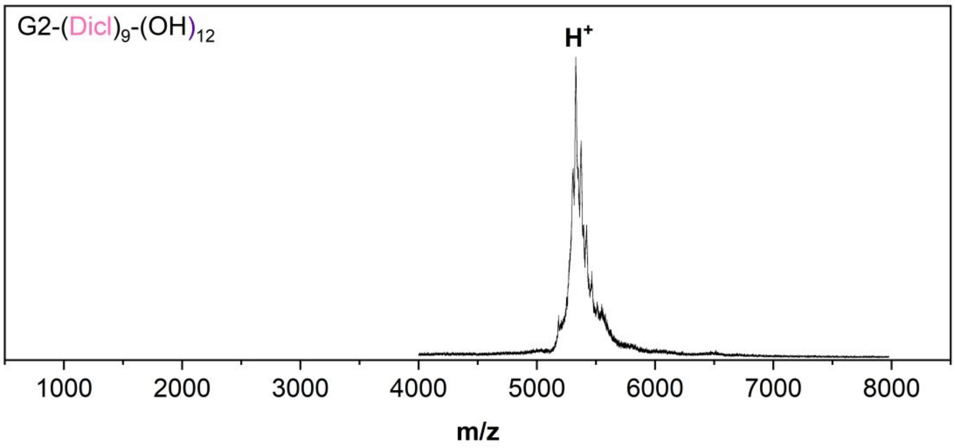

Figure S13. MALDI-TOF spectra of G2-(Dicl)$_9$-(OH)$_{12}$ in DCTB.

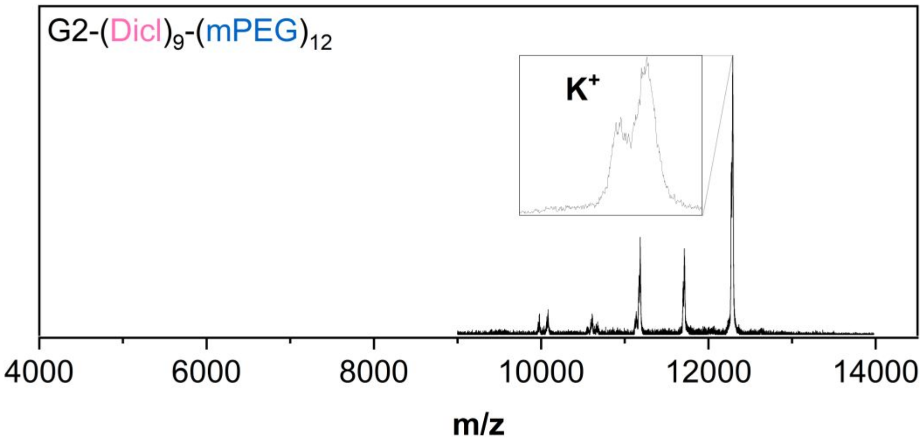

Figure S14. MALDI-TOF spectra of G2-(Dicl)$_9$-(mPEG)$_{12}$ in DCTB.

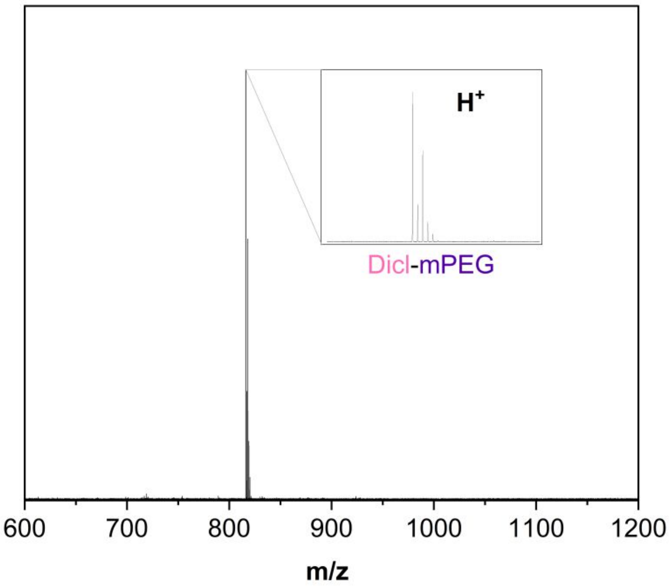

Figure S15. MALDI-TOF spectra of Dicl-mPEG in DHB.

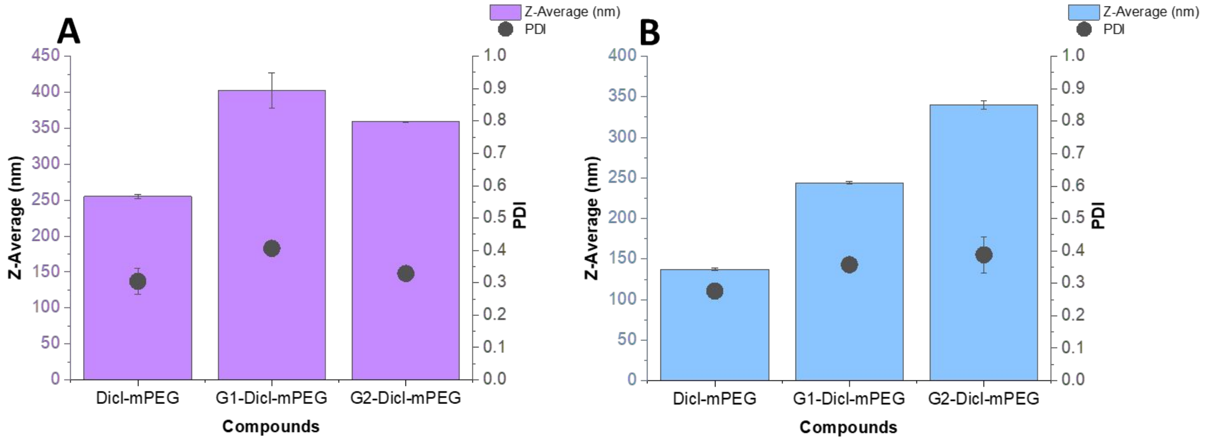

C

DLS: Size evaluation at 40 µM

	Z-Average (nm)	D_i (nm)	D_v (nm)	D_n (nm)	PDI
**Dicl-mPEG**	255.1 ± 2.9	88.9 ± 5.7	83.8 ± 4.7	77.7 ± 3.4	0.30 ± 0.04
**G1-(Dicl)$_3$-(mPEG)$_6$**	402.2 ± 24.9	160.1 ± 5.5	160.2 ± 6.0	4.6 ± 0.3	0.40 ± 0.02
**G2-(Dicl)$_9$-(mPEG)$_{12}$**	358.9 ± 0.6	353.2 ± 12.1	360.5 ± 13.2	332.0 ± 9.0	0.32 ± 0.02

D

DLS: Size evaluation at 500 µM

	Z-Average (nm)	D_i (nm)	D_v (nm)	D_n (nm)	PDI
**Dicl-mPEG**	137.4 ± 1.8	111.3 ± 5.8	96.7 ± 3.4	79.2 ± 3.2	0.27 ± 0.001
**G1-(Dicl)$_3$-(mPEG)$_6$**	243.9 ± 1.6	227.8 ± 13.0	230.9 ± 13.3	5.5 ± 0.7	0.35 ± 0.01
**G2-(Dicl)$_9$-(mPEG)$_{12}$**	339.7 ± 5.3	460.1 ± 34.6	520.5 ± 54.1	215.9 ± 190.3	0.38 ± 0.05

Figure S16. DLS analysis of Dicl-mPEG, G1-(Dicl)$_3$-(mPEG)$_6$ and G2-(Dicl)$_9$-(mPEG)$_{12}$ at 37 °C. Z-average size (nm, bars, left axis) and polydispersity indices (circles, right axis) at (A) 40 µM and (B) 500 µM. Summary of hydrodynamic diameters for representative constructs, including intensity- (D_i), volume- (D_v), and number-weighted (D[illegible]) diameters at (C) 40 µM and (D) 500 µM. Mean values accompanied by standard deviation (SD), n ≥ 3.

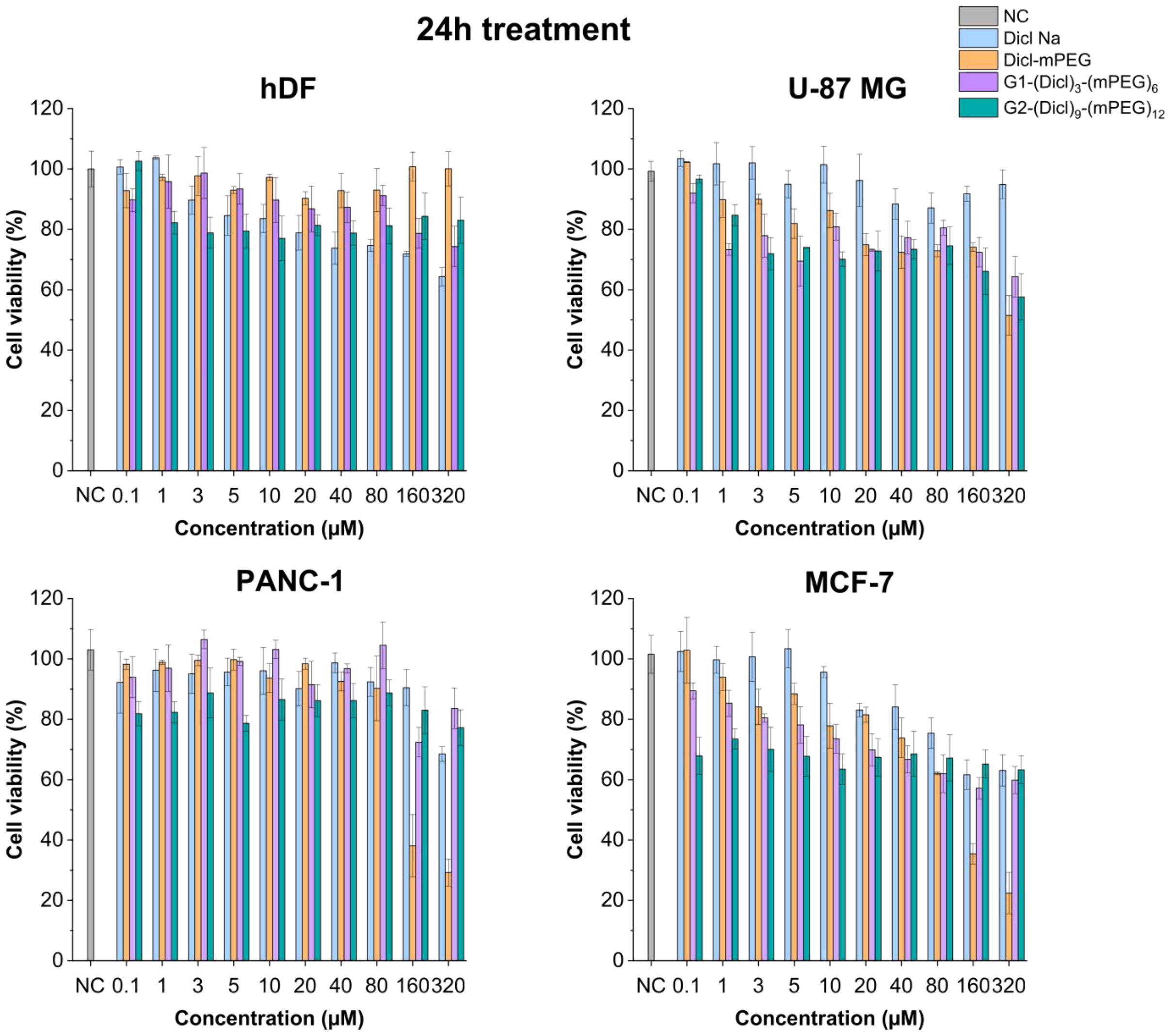

Figure S17. Cytotoxicity evaluation of Dicl-Na, Dicl-mPEG, G1-(Dicl)$_3$-(mPEG)$_6$ and G2-(Dicl)$_9$-(mPEG)$_{12}$ in hDF, U-87 MG, PANC-1 and MCF-7 cell lines after 24h treatment. Data are presented as mean ± SD (n = 3).

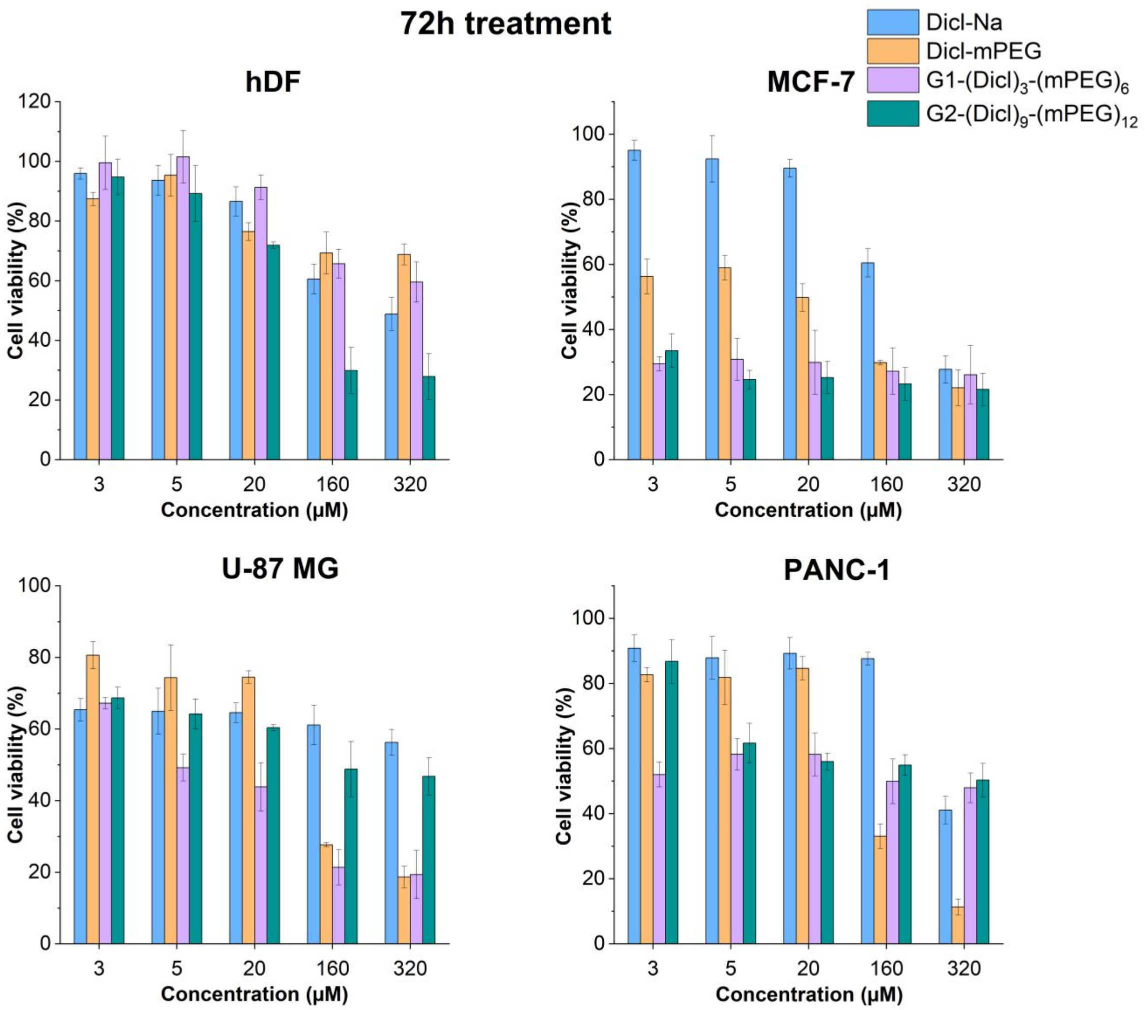

Figure S18. Cytotoxicity evaluation of Dicl-Na, Dicl-mPEG, G1-$(Dicl)_3$-$(mPEG)_6$, and G2-$(Dicl)_9$-$(mPEG)_{12}$ in hDF, MCF-7, U-87 MG, and PANC-1 cell lines after 72 hours of treatment at concentrations of 3 µM to 320 µM. Data are presented as mean ± SD (n = 3).

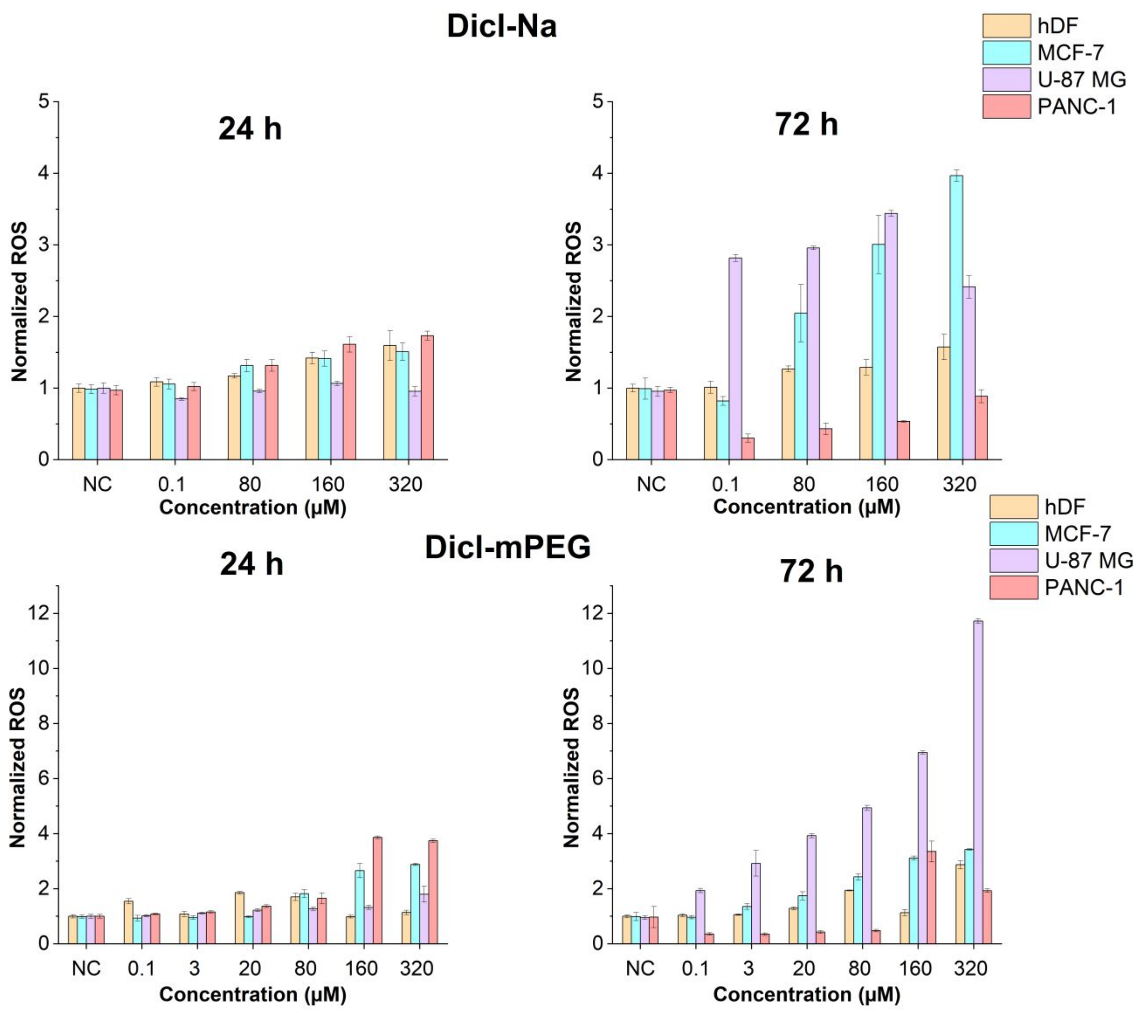

Figure S19. Time- and concentration-dependent ROS generation induced by (A) Dicl-Na and (B) Dicl-mPEG at 24 and 72 h across hDF, U-87 MG, PANC-1, and MCF-7 cell lines. Data was normalized to untreated controls and reported as mean ± SD (n = 2-3).

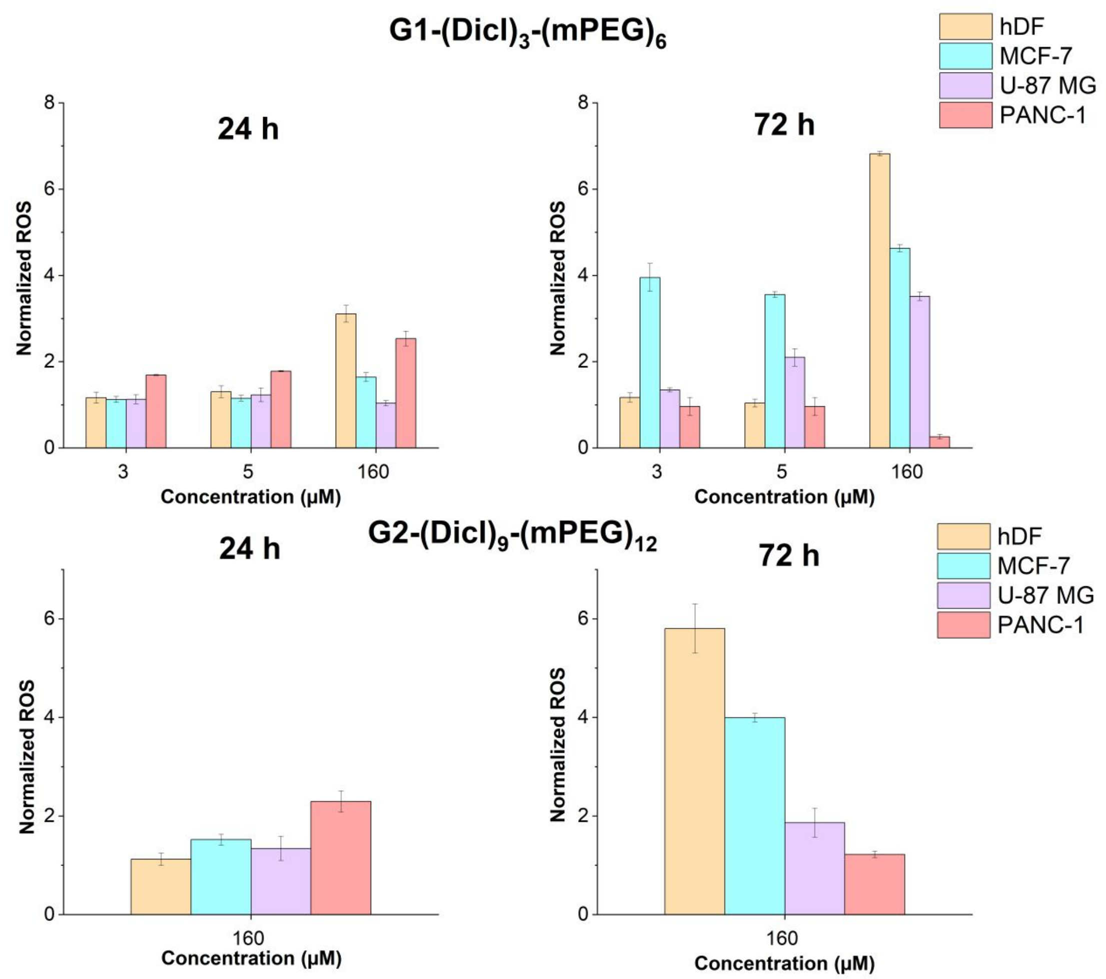

Figure S20. Time- and concentration-dependent ROS generation induced by (A) G1-(Dicl)$_3$-(mPEG)$_6$ (3-160 µM) and (B) G2-(Dicl)$_9$-(mPEG)$_{12}$ (160 µM) at 24 and 72 h across hDF, MCF-7, U-87 MG, and PANC-1 cell lines. Data was normalized to the percentage of viable cells obtained from the cytotoxicity assays and reported as mean ± SD (n = 2-3).

References

1. Singh, A.; Hutchinson, D. J.; Montañez, M. I.; Sanz Del Olmo, N.; Malkoch, M. Synthesis, Evaluation, and Modification of Heterofunctional Polyester Dendrimers with Internally Queued Bromide Groups. *Soft Matter* **2024**, *20* (38), 7573–7577.
2. Y. Zhang, P. Mesa-Antunez, L. Fortuin, O. C. J. Andrén and M. Malkoch, *Biomacromolecules* **2020**, 21, 4294-4301.
